# A functional interaction between liprin-α1 and B56γ regulatory subunit of protein phosphatase 2A supports tumor cell motility

**DOI:** 10.1038/s42003-022-03989-3

**Published:** 2022-09-28

**Authors:** Marta Ripamonti, Andrea Lamarca, Norman E. Davey, Diletta Tonoli, Sara Surini, Ivan de Curtis

**Affiliations:** 1 San Raffaele Scientific Institute and Università Vita-Salute San Raffaele, Milano, Italy; 2 Division of Cancer Biology, The Institute of Cancer Research, 237 Fulham Road, London, SW3 6JB UK

**Keywords:** Focal adhesion, Cell invasion

## Abstract

Scaffold liprin-α1 is required to assemble dynamic plasma membrane-associated platforms (PMAPs) at the front of migrating breast cancer cells, to promote protrusion and invasion. We show that the N-terminal region of liprin-α1 contains an LxxIxE motif interacting with B56 regulatory subunits of serine/threonine protein phosphatase 2A (PP2A). The specific interaction of B56γ with liprin-α1 requires an intact motif, since two point mutations strongly reduce the interaction. B56γ mediates the interaction of liprin-α1 with the heterotrimeric PP2A holoenzyme. Most B56γ protein is recovered in the cytosolic fraction of invasive MDA-MB-231 breast cancer cells, where B56γ is complexed with liprin-α1. While mutation of the short linear motif (SLiM) does not affect localization of liprin-α1 to PMAPs, localization of B56γ at these sites specifically requires liprin-α1. Silencing of B56γ or liprin-α1 inhibits to similar extent cell spreading on extracellular matrix, invasion, motility and lamellipodia dynamics in migrating MDA-MB-231 cells, suggesting that B56γ/PP2A is a novel component of the PMAPs machinery regulating tumor cell motility. In this direction, inhibition of cell spreading by silencing liprin-α1 is not rescued by expression of B56γ binding-defective liprin-α1 mutant. We propose that liprin-α1-mediated recruitment of PP2A *via* B56γ regulates cell motility by controlling protrusion in migrating MDA-MB-231 cells.

## Abstract (web summary)

Liprin-α1 interacts with and recruits B56γ-PP2A at the front of migrating breast cancer cells, to promote cell protrusion and motility.

## Introduction

The serine/threonine protein phosphatase 2A (PP2A) regulates many important cellular processes^1–3^, including adhesion, migration, and focal adhesion dynamics^4^, which are relevant for tumor cell invasion. PP2A holoenzymes are formed by a heterodimeric core complex including the catalytic C and scaffolding A subunits (PP2A-C/A) that associates to one of several B regulatory subunits to direct the holoenzyme to specific intracellular sites and substrates. There are several regulatory subunits for PP2A that belong to four families, each including different isoforms: B/B55, B′/B56/PR61, B′′/PR72, B′′′/PR93^5^. The role of PP2A in cancer is unclear; PP2A is often considered a tumor suppressor, but may also play a positive role in the formation of metastases^6^. The investigation of the molecular mechanisms that underlie the regulation of PP2A by different regulatory subunits in tumor cells is therefore important to understand the distinct roles of this phosphatase in cancer.

It has been recently reported by mass spectrometry and in silico-based proteomic analyses that the PP2A regulatory B56 subunits bind with high specificity to short linear motifs (SLiMs) characterized by the consensus sequence L/MxxI/LxE that is found in several B56/PP2A protein ligands^7^. SLiMs are a class of compact functional interfaces involved in specific protein-protein interactions that are highly enriched in intrinsically disordered regions (IDRs)^8^. Numerous potential B56-binding SLiMs have been identified by in silico analysis including an instance in the liprin-α family of scaffold proteins^7^. The ubiquitously expressed liprin-α1 has been involved in the regulation of cell adhesion, motility and invasion that are relevant to cancer progression^9^. Liprin-α1 interacts directly with several protein partners and includes polypeptide regions that are predicted to be intrinsically disordered^10^. In cells liprin-α1 is part of a network of scaffold and signaling proteins including the ERC1/ELKS, LL5 adaptors that form dynamic plasma membrane-associated platforms (PMAPs) near the edge of migrating tumor cells^11^. At the cell edge these proteins regulate motility, invasion and focal adhesion dynamics^12–14^. Previous proteomic analyses have shown the interaction of the mammalian and *Drosophila* B56 regulatory subunits with liprin-α proteins^15,16^. The human B56 family has five closely related members^6^. In this study we have identified a SLiM in the N-terminal IDR of liprin-α1 that is required for the specific binding to B56γ. Moreover, we show that this SLiM-mediated interaction guides the binding of liprin-α1 to the PP2A holoenzyme. Liprin-α1 is required for the recruitment of B56γ at PMAPs, and point mutations of the liprin-α1 SLiM impair tumor cell motility. Our results show that liprin-α1 recruits B56γ-PP2A at PMAPs near focal adhesions at the front of migrating tumor cells, where PP2A phosphatase activity may influence the turnover of phosphorylated proteins to promote protrusion.

## Results

### Liprin-α1 interacts *via* the N-terminal SLiM with the B56γ regulatory subunit of PP2A

**Fig. 1:**
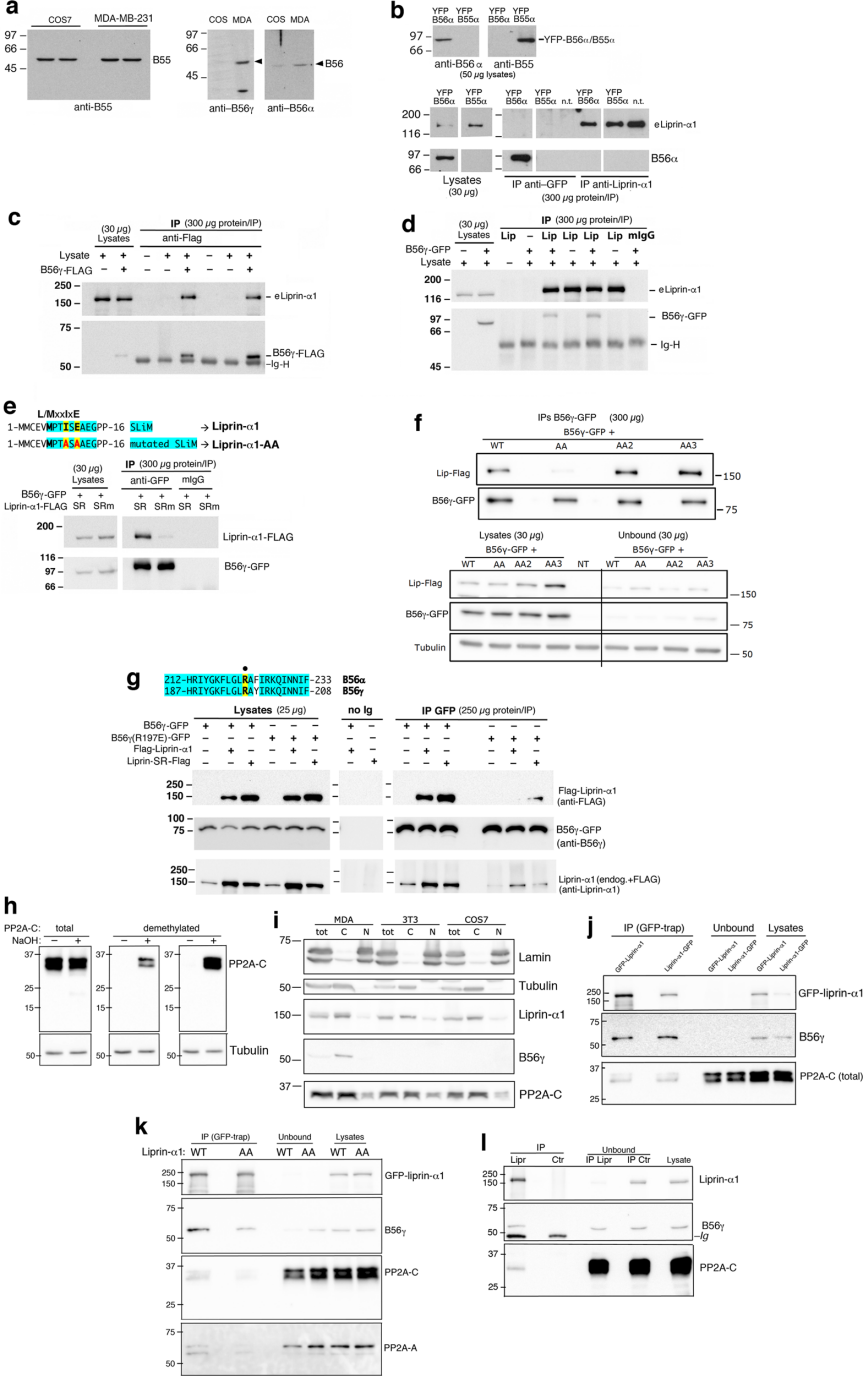
The interaction between liprin-α1 and B56γ-PP2A requires the N-terminal SLiM of liprin-α1, and the SLiM binding pocket of B56γ. **a** Lysates of COS7 and MDA-MB-231 cells (50 µg/lane) blotted with B55 or B56 isoform–specific Abs. **b** Lysates of COS7 cells transfected with YFP-B56α or YFP-B55α were immunoprecipitated with anti-GFP or anti-liprin-α1 Abs, and immunoblotted to reveal the indicated antigens (eliprin-α1, endogenous liprin-α1). **c** Immunoprecipitates with anti-FLAG from lysates of COS7 cells transfected with B56γ-FLAG were blotted for liprin-α1 and B56γ; mIgG, control non-immune mouse IgG. **d** Lysates from COS7 cells transfected with B56γ-GFP immunoprecipitated with anti-liprin-α1 Ab, non-immune mouse IgG (mIgG), or no Ab (–), and blotted with anti-liprin-α1 and anti-GFP Abs. **e** Top: alignment of N-terminus of human wildtype (liprin-α1) and mutant (liprin-α1-AA, with two amino acid substitutions within the SLiM). Bottom: lysates of COS7 cells transfected with B56γ-GFP alone, or together with either liprin-α1 -FLAG or liprin-α1-AA-FLAG, were immunoprecipitated with anti-GFP or control IgG (mIgG), and blotted to reveal siRNA resistant wildtype (WT) and mutant (AA) FLAG-liprin-α1, and B56γ-GFP. **f** Lysates of COS7 cells cotransfected with B56γ-GFP and either wildtype (WT) or SLiM–mutated liprin-α1-FLAG (AA, AA2, AA3) were immunoprecipitated with anti-GFP, and blotted to reveal siRNA resistant wildtype (WT) and mutant (AA, AA2, AA3) liprin-α1-FLAG, and B56γ-GFP. NT, control lysate from non-transfected cells. **g** Top: sequence alignment of B56α and B56γ: in yellow the mutated arginine residue: B56α-R222E and B56γ-R197E. Bottom: lysates from COS7 cells transfected with either B56γ-GFP or B56γ-R197-GFP, or cotransfected with B56γ-GFP and FLAG-tagged liprin-α1, were immunoprecipitated with anti-GFP (no Ig = control beads). Immunoprecipitates and lysates were blotted to reveal FLAG-tagged liprin-α1 (top), and B56γ-GFP (center). The top filter reprobed with anti-liprin-α1 reveals both endogenous and FLAG-liprin-α1. **h** The endogenous catalytic PP2A-C subunit in MDA-MB-231 cells is methylated. Filters with MDA-MB-231 cell lysates (30 µg/lane) untreated (–) or treated with NaOH (+) were incubated with Ab against the central part of the PP2A-C polypeptide recognizing both methylated and demethylated PP2A-C (total), or with two distinct Abs specific for demethylated PP2A-C. **i** Total (tot), cytosolic (C) and nuclear (N) fractions from different cell types were analyzed by immunolotting with the indicated Abs. **j** GFP-liprin-α1 interacts with the PP2A holoenzyme via B56γ. Immunoprecipitations (GFP-Trap) from 100 µg of protein lysate; lysates and unbound fractions, 10 µg protein/lane. **k** Mutation of the SLiM reduces the interaction of liprin-α1 with the B56γ-PP2A holoenzyme. Immunoprecipitations (GFP-Trap) from 300 µg of protein lysate; lysates and unbound fractions, 30 µg protein/lane. **l** Endogenous complex between liprin-α1 and PP2A in MDA-MB-231 cells. Immunoprecipitation (200 µg of protein lysate) of endogenous liprin-α1 (IP Lipr) pulls down catalytic and regulatory subunits of endogenous PP2A. IP Ctr, control immunoprecipitation with mouse Ig; 40 µg/lane of unbound fractions and lysate.

The PP2A holoenzyme is a heterotrimer formed by the PP2A-C catalytic subunit, the PP2A-A structural subunit, and one of several B regulatory subunits. Based on a previous in silico screening^7^, we have identified a new N-terminal SLiM (^6^MPTISE^11^) in human liprin-α1 that may be recognized by B56, but not by B55 regulatory subunits. Breast cancer MDA-MB-231 cells express B56α and B56γ regulatory subunits (Fig. 1a). At first, the interaction of overexpressed B56α and B55α with liprin-α1 was tested by immunoprecipitation in COS7 cells. Immunoprecipitation with anti-GFP antibodies of either YFP-B56α or YFP-B55α failed to co-immunoprecipitate endogenous liprin-α1. Also, reciprocal immunoprecipitation of endogenous liprin-α1 failed to co-immunoprecipitate YFP-B56α and YFP-B55α (Fig. 1b). Conversely, COS7 lysates positive for either B56γ-FLAG or B56γ-GFP (B56γ_3_ isoform) and subjected to immunoprecipitation with either anti-liprin-α1 or anti-GFP, showed a clear interaction of endogenous liprin-α1 with B56γ (Fig. 1c, d). We expect that the interaction between B56γ and liprin-α1 is prevented by mutations in the SLiM of liprin-α1. We prepared siRNA-resistant (sr) sr-liprin-α1-AA mutant carrying two mutations (Ile→Ala and Glu→Ala) in the N-terminal SLiM (^6^**M**PT**I**S**E**^11^ **→** ^6^**M**PT**A**S**A**^11^ = mutant liprin-α1-AA). We used coimmunoprecipitation with either wildtype or liprin-α1 carrying the mutant SLiM to show that these mutations were sufficient to strongly reduce the interaction between liprin-α1-AA and B56γ (Fig. 1e). The efficient interaction between B56γ and liprin-α1 required an intact N-terminal SLiM of the sort identified to interact with B56^7^.

Two more L/MxxI/LxE SLiMs have been identified in liprin-α1 in a peptidome screening for possible B56γ binding partners; the two SLiMs correspond to residues 51-59 (SLIM2) and residues 1081-1089 (SLIM3) of liprin-α1^17^. To test whether these SLiMs contribute to the interaction of liprin-α1 with B56γ, we prepared SLiM mutants by introducing two mutations at the 4th and 6th residues of each SLiM. We prepared sr sr-liprin-α1-AA2 and sr-liprin-α1-AA3 mutants carrying mutations Leu→Ala and Glu→Ala either in the second SLiM (^51^**L**DT**L**R**E**TQE^59^ → ^51^**L**DT**A**R**A**TQE^59^), or in the third SLiM (^1081^**L**LA**L**D**E**TFD^1089^ **→** ^1081^**L**LA**A**D**A**TFD^1089^). We used coimmunoprecipitation with either wildtype or liprin-α1 carrying one of the three mutant SLiMs. We found that while the interaction of liprin-α1-AA with B56γ was strongly inhibited, no evident effects were observed on the interaction of B56γ with either liprin-α1-AA2 or liprin-α1-AA3 (Fig. 1f). Thus, ^6^**M**PT**I**S**E**^11^ is the SLiM responsible for the interaction of liprin-α1 with B56γ.

SLiMs are recognized by grooves in globular domains of their binding partners^18^. A number of B56α binding partners containing L/MxxIxE SLiMs share a specific binding pocket in B56α. An evolutionary/structural analysis and structural information of the human B56γ proteins (https://www.rcsb.org/structure/5SW9) revealed the existence of a well-conserved, surface-exposed pocket on B56, with features that could accommodate binding of L/MxxIxE motifs^7^. Accordingly, mutation analysis shows that Arg-222 is one residue within the pocket required for the interaction of B56α with its binding partners, since mutation of arginine 222 residue to glutamine (R222E) strongly reduced the binding of B56α to partners like separase, KIF4A, BubR1 and GEF-H1, without affecting the binding of B56α to the catalytic and scaffolding subunits of the PP2A^7^. We tested whether the mutation of the corresponding conserved residue in human B56γ (R197E) could affect the binding to liprin-α1. Co-immunoprecipitation from lysates of COS7 cells cotransfected with liprin-α1 and either wildtype or mutant B56γ showed the importance of the conserved positive residue in position 197, as a strong reduction of binding to liprin-α1 was observed upon Arg-to-Glu mutation (Fig. 1g).

Thus liprin-α1 interacts with B56γ, and this interaction is inhibited by mutation of the N-terminal SLiM in the liprin-α1-AA mutant. The results indicate that both the N-terminal MxxIxE SLiM of liprin-α1 and the M/LxxIxE binding pocket of B56γ are required for the efficient interaction between B56γ and liprin-α1.

### B56γ mediates the interaction of liprin-α1 with the PP2A heterotrimeric holoenzyme

Methylation of the C-terminal leucine 309 residue of catalytic PP2A-C is crucial for the interaction with the regulatory subunit required to assemble the functional holoenzyme, including B56γ-containing holoenzymes^19–22^. We determined the methylation state of PP2A-C in MDA-MB-231 cells. Notably, PP2A-C was virtually fully methylated in MDA-MB-231 breast cancer cells, since different Abs recognizing the demethylated catalytic subunit detected PP2A-C in MDA-MB-231 cell lysates only after demethylation by alkaline hydrolysis (NaOH)^23^, while an Ab against the central region of the subunit recognized the PP2A-C independently of methylation (Fig. 1h).

Overexpressed B56γ has been reported to localize to the nucleus and cytoplasm to regulate specific functions of the PP2A holoenzyme^24^. Interestingly, cell fractionation shows that endogenous B56γ was fully recovered in the cytosolic fraction of MDA-MB-231 cells (Fig. 1i). Based on the results shown in Fig. 1i, we argue that the cytosolic pool of endogenous B56γ could be entirely complexed to liprin-α1. In fact, the immunoprecipitation of either N- or C-terminally GFP-tagged liprin-α1 virtually depleted the endogenous B56γ from unbound fractions (Fig. 1j). We tested if the B56γ regulatory subunit interacting with liprin-α1 was part of the heterotrimeric PP2A holoenzyme. GFP-liprin-α1 from transfected MDA-MB-231 co-precipitated with endogenous B56γ, PP2A-A, and PP2A-C subunits (Fig. 1k), demonstrating that B56γ/PP2A binds to liprin-α1. While most of the endogenous B56γ was in complex with GFP-liprin-α1, a large fraction of the core subunits (PP2A-A and PP2A-C) remained in the unbound fraction. This result indicates that the B56γ-containing hetero-complexes bound efficiently to liprin-α1. The PP2A-A/C heterodimers left in the unbound fraction are likely available for the interaction with other regulatory subunits. Importantly, immunoprecipitation of the mutant GFP-liprin-α1-AA reduced the binding of endogenous B56γ as well as of PP2A-A and PP2A-C (Fig. 1k), suggesting that B56γ is the main regulatory subunit mediating the interaction of liprin-α1 with the PP2A holoenzyme in MDA-MB-231 tumor cells. These data are consistent with the concomitant expression in these cells of different B regulatory subunits^25^ that may interact with distinct subpopulations of the dimeric PP2A-A/C core heterodimers. In addition, immunoprecipitation of endogenous liprin-α1 revealed the endogenous complex of liprin-α1 with the PP2A holoenzyme (Fig. 1l).

Liprin-α1 and B56γ proteins were expressed by a number of different breast cancer cell lines (MCF-7, BT-474, SK-BR-3, T-47D) differing in terms of molecular phenotypes and metastatic potential (Supplementary Fig. 1a). Importantly, the endogenous complex including liprin-α1, B56γ and the catalytic PP2A-C subunit could be detected in all cell lines tested (Supplementary Fig. 1b). As for MDA-MB-231 cells (Fig. 1h), we found that the catalytic PP2A-C was virtually completely methylated (i.e., suitable for the active state) in all cell lines analyzed here (Supplementary Fig. 1c). These data argue for a widespread function of this interaction across different breast cancer cell types.

Overall, these results show the interaction of B56γ-containing PP2A holoenzymes, which is dependent on the SLiM detected at the N-terminus of liprin-α1.

### Liprin-α1 recruits B56γ at PMAPs in migrating MDA-MB-231 cells

The specific localization of liprin-α1 at PMAPs and the interaction of B56γ with liprin-α1 suggest a possible liprin-α1-dependent accumulation of B56γ at PMAPs. No antibodies are available to detect the subcellular localization of endogenous B56γ. Colocalization of B56γ-GFP with endogenous liprin-α1 at PMAPs was evident at the front of migrating MDA-MB-231 cells, and colocalization was sometimes less evident for the liprin-defective mutant B56γ^R197E^-GFP (Supplementary Fig. 2a). The localization of liprin-α1 was not affected by mutation of the B56γ–binding SLiM: both wildtype liprin-α1 and liprin-α1-AA colocalized with endogenous ERC1 at PMAPs near protrusions of migrating MDA-MB-231 (Supplementary Fig. 2b). B56γ-GFP colocalized with liprin-α1-FLAG at PMAPs, while the colocalization of B56γ^R197E^-GFP with the binding-defective mutant liprin-α1-AA was less pronounced (Supplementary Fig. 2c).

**Fig. 2:**
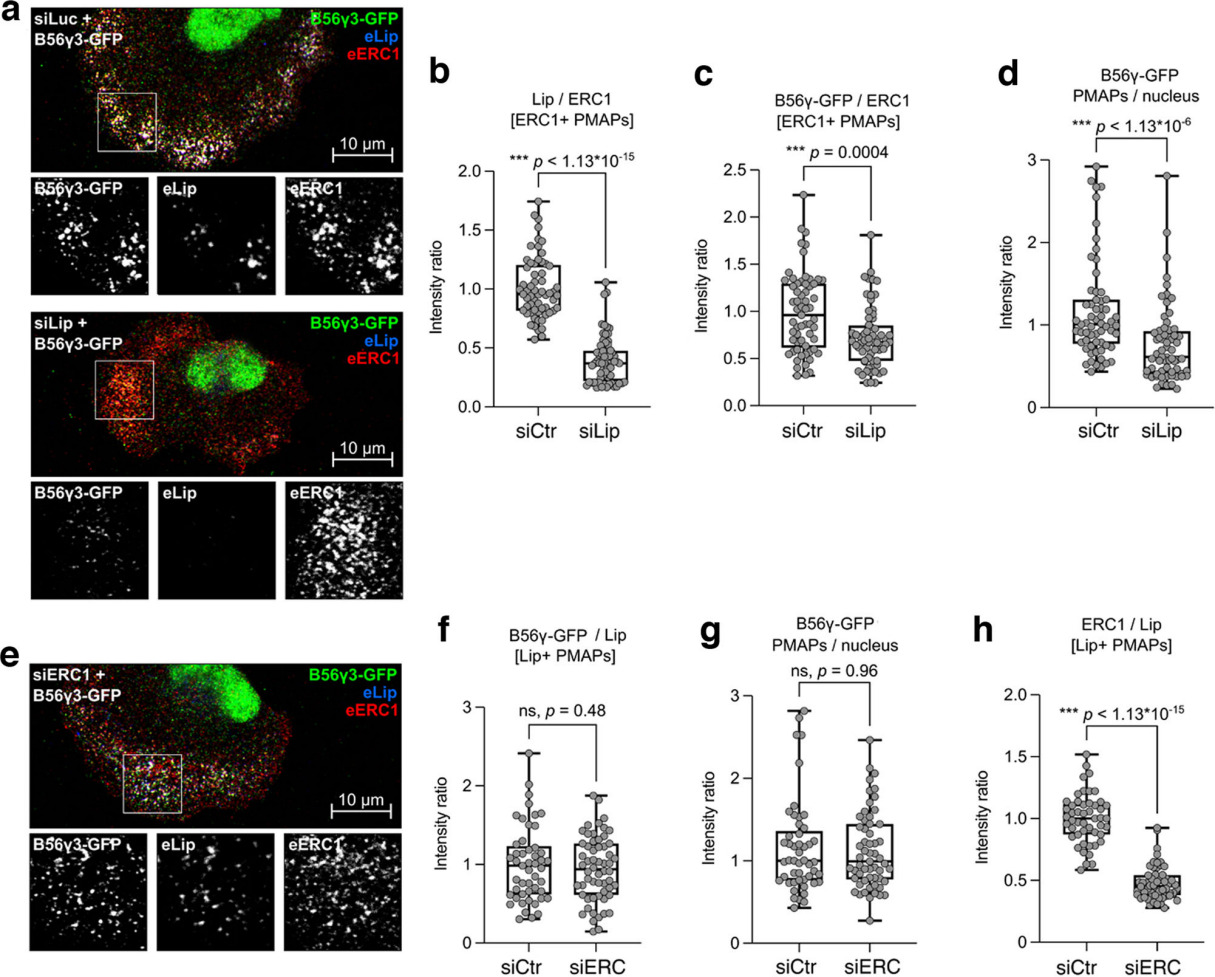
Liprin-α1 directs cytoplasmic B56γ at PMAPs. MDA-MB-231 cells expressing B56γ-GFP treated with saponin and fixed with PFA and immunostained. **a**–**d** Accumulation of B56γ-GFP in ERC1-positive PMAPs, in the presence of control siRNA (siCtr) or anti-liprin-α1 siRNA (siLip). **a** Representative confocal images. **b** Quantification of the liprin-α1-derived fluorescence in ERC1-positive PMAPs, expressed as Liprin-α1/ERC1 ratio, revealed efficient silencing of Liprin-α1 in cells cotransfected with B56γ-GFP and siLip. **c** Quantification of B56γ-GFP in ERC1-positive PMAPs, represented as a ratio of the intensity of the two proteins, as revealed by immunofluorescence. **d** Quantification of B56γ-GFP signal in PMAPs in respect to its expression level, as determined by the fluorescence in the nucleus. **e**–**h** Accumulation of B56γ-GFP in Liprin-α1-positive PMAPs, in the presence of control siRNA (siCtr) or anti-ERC1 siRNA (siERC1). **e** Representative confocal images. **f** Quantification of B56γ-GFP in liprin-α1-positive PMAPs, represented as a ratio of the intensity of the two proteins, as revealed by immunofluorescence. **g** Quantification of the B56γ-GFP signal in PMAPs in respect to its expression level, as determined by the fluorescence in the nucleus. **h** Quantification of the ERC1-derived fluorescence in liprin-α1-positive PMAPs, expressed as ERC1/liprin-α1 ratio, revealed efficient silencing of ERC1 in cells cotransfected with B56γ-GFP and siERC1. eLip, endogenous liprin; eERC1, endogenous ERC1; the same contrast was applied to confocal images in (**a** and **e**).

Although expression of B56γ-GFP could show localization of this protein at PMAPs in migrating tumor cells, images were not suitable for quantitative analysis due to the diffuse cytoplasmic signal possibly caused by the high level of expression of B56γ-GFP. We mildly permeabilized cells with a low concentration of saponin^26^ to remove the excess of B56γ-GFP and highlight the specific binding to liprin-α1-positive/ERC1-positive PMAPs. The localization of B56γ-GFP at PMAPs relied on the specific binding to endogenous liprin-α1: MDA-MB-231 cells transfected with B56γ-GFP combined with either control siRNA (siCtr) or siRNA against liprin-α1 (siLip) (Supplementary Fig. 3a) were imaged by confocal microscopy (Fig. 2a, Supplementary Fig. 3b). Although liprin-α1 silencing negatively affects the formation of ERC1-positive PMAPs^13^, a population of cells presenting some ERC1 accumulation at protrusions was still present due to incomplete depletion of liprin-α1 (Supplementary Fig. 3a). The drastic reduction of the liprin-α1/ERC1 intensity ratio in ERC1-positive PMAPs confirmed the efficient downregulation of liprin-α1 (Fig. 2b, Supplementary Fig. 3b). The mean fluorescence intensity ratio of B56γ-GFP in ERC1-positive PMAPs dropped to 60% after liprin-α1 silencing (Fig. 2c). To compensate for the variability in the expression levels of B56γ-GFP in different cells, we normalized the intensity of B56γ-GFP in PMAPs to its nuclear signal: a strong decrease of fluorescence intensity ratio (B56γ-GFP PMAPs/nucleus) was observed after liprin-α1 silencing (Fig. 2d). Since the signal of nuclear B56γ-GFP was unchanged after liprin-α1 silencing compared to control cells (Supplementary Fig. 3c), the results support the conclusion that liprin-α1 silencing causes a specific loss of B56γ-GFP from ERC1-positive PMAPs.

The fluorescence intensity within liprin-α1-positive PMAPs did not significantly differ between control and ERC1 silenced cells (Fig. 2e, f, Supplementary Fig. 3a, d), neither did the B56γ-GFP PMAPs/nucleus intensity ratio (Fig. 2g, Supplementary Fig. 3e). As expected, the ERC1/liprin-α1 ratio dropped strongly in PMAPs of ERC1 silenced cells compared to control cells (Fig. 2h)^13^. We conclude that liprin-α1 guides cytosolic B56γ-GFP at PMAPs, suggesting that liprin-α1 directs the PP2A holoenzyme’s activity toward the edge of migrating tumor cells.

### B56γ is required for efficient cancer cell motility and invasion

**Fig. 3:**
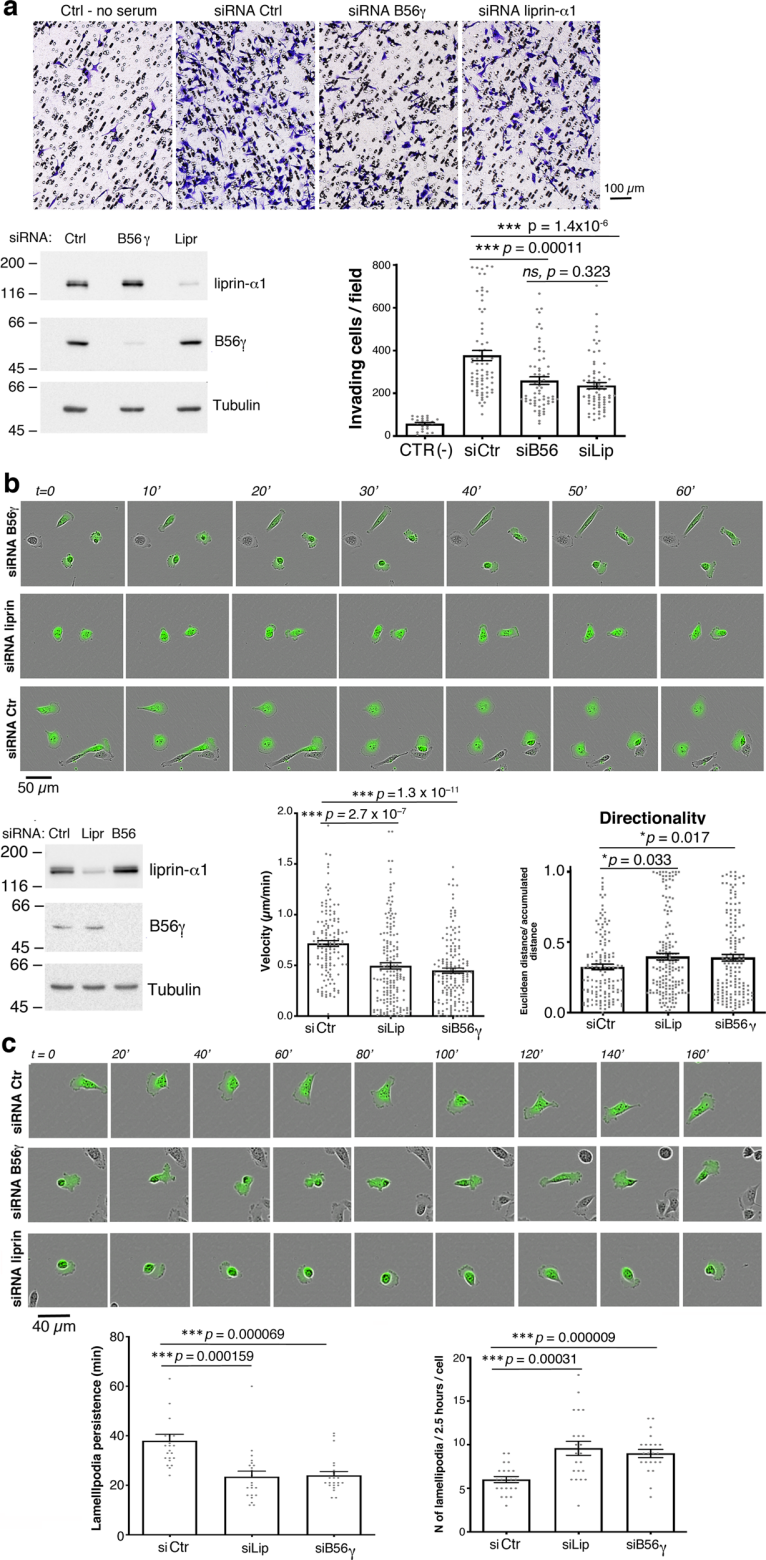
B56γ is required for efficient tumor cell migration and invasion. **a** Silencing of either B56γ or liprin-α1 inhibits Matrigel^TM^ invasion by MDA-MB-231 cells. Top, fields from wells with invading cells. Bottom: left, immunoblotting from lysates of siRNA-transfected cells used for invasion (50 µg protein/lane); right: bars represent the average number of invading cells per field; *n* = 64–71 fields from 10 to 12 wells, from 4 experiments; *n* = 24 fields from 4 wells for control (no stimulus). **b** Silencing of either B56γ or liprin-α1 inhibits tumor cell migration. Top, frames from time-lapses of GFP-positive MDA-MB-231 cells transfected with siRNAs. Bottom: left, immunoblotting from lysates of cells used for random migration (50 µg protein/lane). Right: average speed and directionality of migrating cells; *n* = 139–167cells/experimental condition from 2 experiments. **c** Silencing of either B56γ or liprin-α1 inhibits lamellipodia dynamics. Top, frames from time-lapses of GFP-positive MDA-MB-231 cells from experiments shown in **b**. Bottom: average persistence of lamellipodia (left; *n* = 22 cells, 137–211 lamellipodia analyzed from 2 experiments) and frequency (right; *n* = 22, from 2 experiments). Graph bars: mean and SE.

Liprin-α1 supports tumor cell motility by promoting focal adhesion and invadosome dynamics^12,14,27^ and the formation of metastases by breast cancer cells^28^. We have tested whether B56γ/PP2A is part of the molecular machinery underlying the liprin-α1-mediated control of cancer cell motility. First, we addressed the effects of B56γ depletion on the invasive potential of MDA-MB-231 in vitro. B56γ targeting siRNA^29,30^ efficiently silenced both endogenous and overexpressed B56γ proteins in MDA-MB-231 cells (Supplementary Fig. 4). Matrigel invasion was significantly inhibited by B56γ silencing (Fig. 3a). Interestingly, this effect was comparable to that observed after liprin-α1 depletion^12^. We analyzed the requirement of B56γ for tumor cell motility. By employing a 2D random migration assay we observed that the inhibitory effects on cell velocity and directionality observed by liprin-α1 depletion were phenocopied by silencing endogenous B56γ (Fig. 3b; Supplementary Movies 1–3). In both cases, the reduced persistence and the increased frequency of formation of lamellipodia could underlie the observed defect in motility (Fig. 3c).

In addition to cell motility, tumor cell invasion requires extracellular matrix degradation by proteases secreted at invadosomes^31^. Endogenous liprin-α1-positive PMAPs form near invadosomes in Src-transformed NIH 3T3 cells (NIH-Src) and in MDA-MB-231 cells expressing a constitutively active c-Src–Y527F mutant (MDA-MB-231-Src)^27^. Interestingly, we observed the accumulation of B56γ-GFP at liprin-α1-positive PMAPs in MDA-MB-231-Src (Supplementary Fig. 5a). Silencing of endogenous B56γ in MDA-MB-231-Src cells did not affect extracellular matrix degradation (Supplementary Fig. 5b), nor the formation of invadosomes (Supplementary Fig. 5c).

Hence, silencing of either B56γ or liprin-α1 inhibits MDA-MB-231 tumor cell motility to a similar extent, comparably perturbing lamellipodia dynamics. The data indicate that B56γ is a critical determinant of breast cancer cell motility, and suggest that this PP2A regulatory subunit is a key player of liprin-α1-dependent pathways.

### The interaction between liprin-α1 and B56γ supports MDA-MB-231 cell spreading and focal adhesions formation

**Fig. 4:**
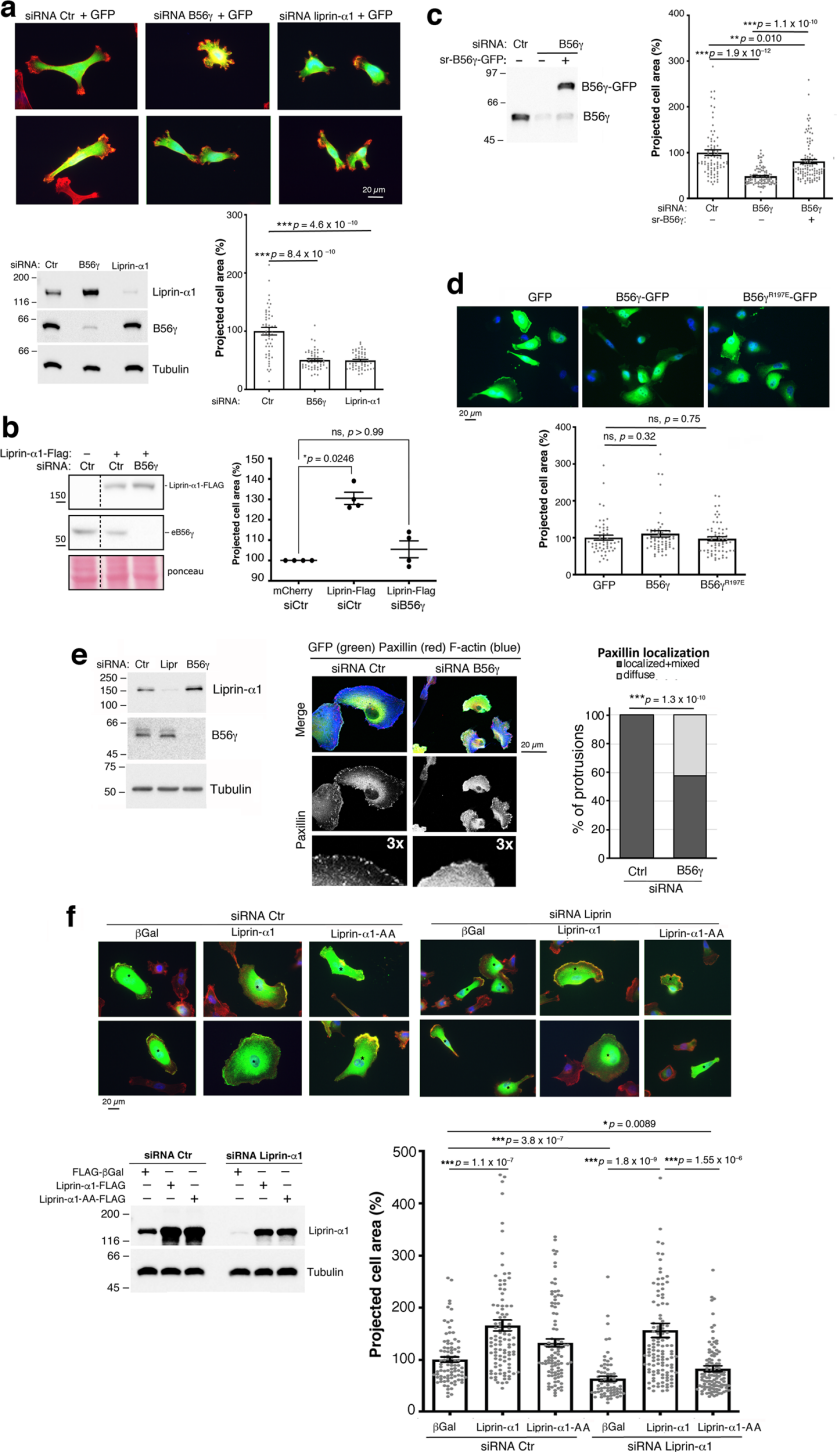
The interaction between liprin-α1 and B56γ promotes MDA-MB-231 cell spreading. **a** Silencing of B56γ and liprin-α1 inhibits MDA-MB-231 cell spreading. Top: transfected cells cultured 18 h on 10 µg/ml fibronectin: GFP (green), F-actin (red), DAPI (blue). Bottom: left, immunoblotting of lysates from siRNA transfected cells (50 µg protein/lane) with indicated Abs; right, quantification of projected cell area (*n* = 52–53 cells); bars: mean and SE. **b** Silencing of endogenous B56γ prevents increase in cell spreading by liprin-α1-FLAG (*n* = 4 experiments). **c** Rescue of cell spreading by expression of sr-B56γ-GFP in cells depleted of endogenous B56γ (*n* = 85–104 cells). **d** Spreading of cells transfected with GFP, B56γ-GFP or B56γ^R197E^-GFP (*n* = 52–64 cells). Cells in (**b**–**d**) were analyzed as in (**a**). Graph bars: mean and SE. **e** Depletion of endogenous B56γ and liprin-α1 by siRNA: 50 µg of protein lysate per lane. Center: confocal images to detect transfected cells (GFP), endogenous paxillin (red) and F-actin (blue). Right: quantification of the localization of endogenous paxillin at focal adhesions (33 and 85 protrusions from cells transfected with either control or B56γ siRNA, respectively); *χ*^2^ test. **f** Spreading of cells cotransfected with siRNAs with either FLAG-βGalactosidase, sr-liprin-α1-FLAG (liprin-α1), or sr-liprin-α1-AA-FLAG (liprin-α1-AA). Graph bars (*n* = 72–116 cells). Graph bars: mean and SE.

Endogenous liprin-α1 is required for efficient integrin-mediated spreading of cells on fibronectin, and liprin-α1 overexpression enhances the spreading of different cell types, including breast cancer MDA-MB-231 cells^12,32^. We found that silencing endogenous B56γ inhibited spreading of MDA-MB-231 cells on fibronectin to the same extent as silencing endogenous liprin-α1 (Fig. 4a). B56γ silencing inhibited also cell spreading enhanced by liprin-α1 overexpression (Fig. 4b). SiRNA-resistant sr-B56γ-GFP could rescue the defect in spreading induced by silencing endogenous B56γ (Fig. 4c). On the other hand, sr-B56γ-GFP overexpression did not enhance cell spreading (Fig. 4d). The results suggest that B56γ is required to support liprin-α1–dependent spreading, and that liprin-α1 is the limiting factor to promote spreading. The expression levels of liprin-α1 influence the morphology and dynamics of focal adhesions in distinct cell types^12,13,32^. We evaluated if silencing B56γ affected the formation of focal adhesions in MDA-MB-231 cells plated on fibronectin (10 µg/ml). Silencing of B56γ interfered with cell spreading and with the localization of endogenous paxillin, which appeared often diffuse at protrusions rather than clearly localized at focal adhesions in siB56γ MDA-MB-231 cells (Fig. 4e).

We finally tested if binding of liprin-α1 to B56γ is required for liprin-α1-mediated cell spreading. Interestingly, while the sr sr-liprin-α1-FLAG was able to rescue the defect in MDA-MB-231 tumor cell spreading induced by silencing endogenous liprin-α1, the sr B56γ binding-defective mutant sr-liprin-α1-AA-FLAG was unable to do so (Fig. 4f), indicating that the interaction of B56γ with liprin-α1 mediated by its N-terminal SLiM is important for the integrin-mediated spreading of tumor cells.

Thus, silencing of either B56γ or liprin-α1 limits MDA-MB-231 cell spreading. The B56γ–binding defective mutant liprin-α1-AA is less efficient than wildtype liprin-α1 in promoting MDA-MB-231 cell spreading, and in rescuing the spreading capacity compromised by silencing endogenous liprin-α1.

## Discussion

The serine/threonine protein phosphatase PP2A is implicated in several cellular events. For this, the catalytic subunit of PP2A is expected to be localized at distinct subcellular sites by interacting with one of several regulatory subunits. Our study has identified the mechanism by which B56γ/PP2A is recruited to the edge of migrating tumor cells, and shows that the interaction of liprin-α1 with B56γ is required for efficient cell spreading, and is likely required for efficient motility of breast cancer cells. We have previously demonstrated that liprin-α1 in human breast cancer cells promotes focal adhesion dynamics, invadosome function, and the formation of metastases^12,14,27^. Here we show that B56γ/PP2A is a new component of the liprin-α1 protein network (Figs. 1, 2). Interestingly, silencing B56γ causes defects in tumor cell motility similar to those observed after silencing liprin-α1 (Fig. 3), supporting the hypothesis that the two proteins are part of the same regulatory machinery. We hypothesize that the recruitment of B56γ/PP2A at PMAPs by liprin-α1 is required to regulate the protrusive activity during tumor cell migration. In support of this hypothesis, disruption of the interaction between the two proteins reduces the capacity of liprin-α1 to support tumor cell spreading (Fig. 4).

An important open question concerns the nature of the molecular mechanism underlying the effects on cell motility observed after interfering with liprin-α1–B56γ interaction. It has been proposed that PP2A limits tumor cell migration by dephosphorylating proteins of the focal adhesions. Pharmacological inhibition of PP2A activity stimulates the migration of endothelial and Lewis lung carcinoma cells, and results in serine hyperphosphorylation of paxillin^33,34^, a focal adhesion protein regulating migration^35^. On the other hand, the use of PP2A inhibitors can simultaneously affect several processes that may require distinct PP2A holoenzymes. By point mutations we specifically addressed the liprin-α1–mediated interaction with B56γ/PP2A: we have highlighted specific effects of the B56γ-liprin-α1 interaction revealed by disrupting one of several possible expected interactions of the B56 regulatory subunits in MDA-MB-231 cancer cells.

Liprin-α1 promotes cancer cell motility by increasing focal adhesion dynamics^13,14^. Since the liprin-α1 protein network includes several focal adhesion proteins^36,37^, it is possible that liprin-α1 binding to B56γ tethers the phosphatase PP2A to focal adhesions, where target proteins of the phosphatase may be found. In this direction, overexpressed B56γ1 localizes to focal adhesions in COS7 cells and interacts with paxillin^38^, which has been suggested to be dephosphorylated on serine residues by PP2A to control Lewis lung carcinoma cell motility^39^. PP2A-induced dephosphorylation of paxillin causes delay in the turnover of focal adhesions and limits malignant progression^4,38^. Although it was previously shown that PP2A-C and B56γ subunits co-immunoprecipitate with paxillin from transfected NIH-3T3 cells^38^, we did not detect any interaction between B56γ and paxillin by immunoprecipitation from migratory MDA-MB-231 breast cancer cells (Supplementary Fig. 6), while B56γ efficiently precipitated endogenous liprin-α1 in these cells. Of note, here we have considered the interaction of paxillin with the B56γ3 isoform, which in the study by Ito and colleagues was found to coprecipitate poorly with paxillin compared to the B56γ1 and B56γ2 isoforms^38^.

Previous identification of a B56γ-liprin-α1 complex distinct from B56γ/PP2A complexes in human embryonic kidney cells led to the hypothesis of a role for B56γ independent of its regulation of PP2A activity^15^. On the other hand, we found that in MDA-MB-231 cells the SLiM-mediated interaction of liprin-α1 with B56γ results in the recruitment of the catalytic and structural subunits to the complex, suggesting that liprin-α1 engages B56γ-containing PP2A heterotrimers at PMAPs.

It is believed that the C-terminal methylation of PP2A-C is important for the formation of stable B56γ/PP2A-C/PP2A-A complexes, including those involved in tumor-suppressive functions^21,40–43^. Interestingly, by employing methylation-sensitive and insensitive anti-PP2A-C Abs, we found that PP2A-C was virtually completely methylated in the highly metastatic MDA-MB-231 breast cancer cell line, and therefore suited for the assembly of B56γ-containing PP2A holoenzymes (Fig. 1h). Of note, many studies have made use of anti-PP2A-C antibodies raised against the recombinant C-terminus of PP2A-C, which we and others have shown to not recognize the methylated PP2A-C. As a result, potentially confusing assumptions concerning the overall activity of PP2A in (tumor) cells have been made^23^. It is intriguing that in MDA-MB-231 tumor cells most endogenous B56γ protein was found in the cytosolic fraction (Fig. 1i), and in complex with endogenous liprin-α1 (Fig. 1l), suggesting that liprin-α1 drives a large fraction of the B56γ/PP2A holoenzyme at PMAPs to regulate tumor cell migration.

One interesting aspect highlighted by our results is the recruitment of B56γ/PP2A at PMAPs, molecular assemblies that may form by liquid-liquid phase separation^11,44^. In addition to liprin-α1, ERC1 and LL5 proteins, PMAPs have been shown to include also the scaffold proteins KANK and liprin-β^36,37^. PMAPs, which have also been referred to as Cortical Microtubule Stabilization Complexes^36^, are dynamic and form during protrusion near the front of migrating breast cancer cells^13^. PMAPs represent a means to dynamically localize protein scaffolds and enzymes like protein kinases and phosphatases to regulate events at the dynamic front of motile cells^45^. Along this line, B56γ localizes to nuclear speckles, a phase separated structure implicated in RNA splicing^46^. Also, PP2A is recruited by RACK1 to phase separated condensates triggered by the interaction of the transcription factor IRF3 with mutants of the tumor suppressor Neurofibromin 2 (NF2), causing neurofibromatosis and multiple malignancies^47^.

Overall, our results suggest a context-dependent function of B56γ-containing PP2A enzyme, previously described as tumor suppressor^43^, and here found to support tumor cell motility. One important issue is to understand how the recruitment of B56γ/PP2A at PMAPs is influencing the protrusive activity of invasive tumor cells. The liprin-α1 recruitment of B56γ/PP2A at PMAPs near focal adhesions may promote their turnover^4^ by altering the phosphorylation state of focal adhesion and/or PMAP components. Alternatively, liprin-α1 may remove PP2A from adhesions to promote invasion.

## Methods

### Plasmids and siRNAs

The plasmid for B56γ_3_-FLAG (referred to as B56γ-FLAG in the Results) was from GenScript. The plasmids for YFP-B55 and YFP-B56α were as described^7^. The plasmid for B56γ3-GFP (referred to as B56γ-GFP in the Results) was obtained by subcloning the cDNA for B56γ3 amplified by PCR from B56γ-FLAG with Phusion^TM^ High-Fidelity DNA Polymerase (BioLabs), and inserted into the pEGFP-N1 vector. Plasmids FLAG–liprin-α1, GFP–liprin-α1, and GFP-sr-liprin-α1 (sr = siRNA-resistant), were as described^13^. The plasmid GFP-sr-liprin-α1-AA was obtained by site-directed mutagenesis from GFP-sr-liprin-α1 using the primers 5′-GGGGCCTTCTGCTGCGCTGGCGGTCGGCATCACC-3′ and 5′-GGTGATGC CGACCGCCAGCGCAGCAGAAGGCCCC-3′. The resulting protein includes the two amino acid substitutions I9A and E11A in the amino–terminal SLiM of human liprin-α1. The plasmids sr-liprin-α1-AA2-FLAG and sr-liprin-α1-AA3-FLAG were obtained by site-directed mutagenesis from sr-liprin-α1-FLAG using the primers 5′-GACCGCCTTCTTGATACAGC GAGAGCGACTCAAGAAACGCTGGC-3′and 5′-GCCAGCGTTTCTTGAGTCGCTCTCG CTGTATCAAGAAGGCGGTC-3′ (for sr-liprin-α1-AA2-FLAG), and 5′-CACGGAGCAC TTCTGGCCGCAGATGCAACCTTCGACTTCAGTGC-3′ and 5′-GCACTGAAGTCGAAG GTTGCATCTGCGGCCAGAAGTGCTCCGTG-3′ (for sr-liprin-α1-AA3-FLAG).

To obtain the sr plasmids sr-liprin-α1-FLAG and sr-liprin-α1-AA-FLAG, the cDNAs for sr-liprin-α1 or sr-liprin-α1-AA were amplified by PCR and inserted into the p3xFLAG-CMV-14 vector. The plasmid B56γ^R197E^-GFP was obtained by site-directed mutagenesis from B56γ-GFP using the primers: 5′-CTGATGTAAGCTTCCAAGCCTAGG AATTTCCCATAGATTCTG-3′ and 5′-CAGAATCTATGGGAAATTCCTAGGCTTGGAA GCTTACATCAG-3′ to introduce the point mutation R197E into SLiM-binding pocket of B56γ. The plasmids sr-B56γ-GFP and sr-B56γ^R197E^-GFP were obtained by site-directed mutagenesis on B56γ-GFP and B56γ^R197E^-GFP respectively, using the primers: 5′-CTGGAAATATTGGGAAGTATAATTAATGGATTCGCATTACCTCTAAAAGAAGAG CACAAGATTTTC-3′ and 5′-GAAAATCTTGTGCTCTTCTTTTAGAGGTAATGCGA ATCCATTAATTATACTTCCCAATAT TTCCAG-3′. The siRNA siB56γ, targeting all three isoforms γ1, γ2 and γ3 of B56γ (targeting sequence: 5′-GGAUUUGCCUUACCACUAA-3′, from Dharmacon), was described previously^29,30^. The plasmid pSGT-Y527F-Src (constitutive active Src) was as previously described^48^.

### Cell culture and transfection

MDA-MB-231 and MCF-7 cells were grown in DMEM/F12 1:1 with 10% fetal bovine serum (FBS), 100 U/ml penicillin, 100 μg/ml streptomycin, 20 mM glutamine. COS7 cells were cultured in DMEM with 10% fetal clone III (Hyclone), 100 U/ml penicillin, 100 μg/ml streptomycin, 20 mM glutamine. NIH-3T3, HeLa (T-REx), BT-474, and SK-BR-3 cells were cultured in DMEM with 10% FBS, 100 U/ml penicillin, 100 μg/ml streptomycin, 20 mM glutamine. TD-47 cells were cultured in RPMI with 10% FBS, 100 U/ml penicillin, 100 μg/ml streptomycin, 20 mM glutamine.

Transient transfections were performed 24 h after seeding cells on plastic or round 13–24 mm diameter glass coverslips using lipofectamine-2000^®^ (Thermo Fisher Scientific, Paisley, UK) and the indicated siRNA (50–100 nM) and/or plasmid (1–6 μg of DNA) for biochemistry or microscopy. Transfection medium (Optimem) was replaced by growth medium 3,5-4 h after transfection. Cells transfected only with plasmids were processed 24–48 h after transfection, while in case of siRNAs (alone or in combination with plasmids) cells were processed 48 h after transfection.

### Biochemical analysis

Cells cooled on ice were washed twice with of ice-cold TBS (150 mM NaCl, 20 mM Tris-HCl pH 7.5), and lysed with 50–150 μl of lysis buffer (0.5% Triton X-100, 150 mM NaCl, 20 mM Tris-Cl pH 7.5, 1 mM NaV, 10 mM NaF, anti-proteases Complete 1× (Roche), 0.5 mM PMSF (Sigma-Aldrich) and 1 mM DTT). After 15 min at 4 °C with rotation or incubation on ice and vortexing every 5 min, the insoluble material was removed by centrifugation at 16,000 RCF for 10 min at 4 °C. Protein concentration in the supernatant lysate was determined using Bradford protein assay (Bio-Rad).

For immunoprecipitation cell lysates were incubated with Protein-A–Sepharose beads (Cytiva), Pierce Protein G Agarose (Thermo Scientific) conjugated to antibodies, GFP-Trap (Chromotek), or anti-FLAG-M2 Affinity Gel (Sigma-Aldrich) before processing for SDS-PAGE and immunoblotting.

For immunoblotting, denatured lysates and immunoprecipitates were separated by SDS-PAGE, and transferred to 0.45 µm PROTRAN^®^ nitrocellulose membranes (GE Healthcare Amersham Biosciences). Membranes were blocked in 5% (w/v) milk diluted in TBST, incubated with primary antibodies, HRP-conjugated secondary antibodies (Table 1), and revealed by Clarity with ChemiDoc MP Imaging System (Bio-Rad). Membranes were reprobed with the indicated antibodies after stripping for 5–10 min at RT with 0.2 M glycine, 0.1% SDS, 1% Tween-20, pH 2.2 and washing at neutral pH. Quantification of protein levels was done with ImageLab software (Bio-Rad).

**Table 1:** Antibodies used in this study.

Primary antibodies						
Target	Antibody name	Supplier	Cat. No.	Type	Host	Comment/Use
PP2A-C	Purified anti-PP2A catalytic α, clone 46	BD Transduction Laboratories^™^	610556	Monoclonal	Mouse	Recognizes methylated and and non-methylated form WB 1:5000
PP2A-C	Anti-demethylated-PP2A-C, Clone 4B7	Santa Cruz Biotechnology	sc-13601	Monoclonal	Mouse	Specific for non-methylated form WB 1:1000
PP2A-C	Anti-PP2A-Cα/β, Clone 1D6	Santa Cruz Biotechnology	sc-13601	Monoclonal	Mouse	Preferential recognition of non- methylated form WB 1:1000
PP2A-C	Anti-PP2A alpha	GeneTex	GTX106334	Polyclonal	Rabbit	Specific for non-methylated form WB 1:5000
B55α	Anti-B55α Clone 2G9	Cell Signaling	5689	Monoclonal	Mouse	WB 1:1000
B56α	Anti-B56α Clone F-10	Santa Cruz	sc-271151	Monoclonal	Mouse	WB 1:100
B56γ	Anti-PP2A-B56γ, Clone E-6	Santa Cruz Biotechnology	sc-374380	Monoclonal	Mouse	WB 1:100-500
PP2A-A	Anti-PP2A-Aα/β Clone 4G7	Santa Cruz Biotechnology	sc-13600	Monoclonal	Mouse	WB 1:250
Calnexin	Purified Mouse Anti-Calnexin	BD Transduction Laboratories^™^	610523	Monoclonal	Mouse	WB 1:2000
Calnexin	Anti-Calnexin antibody produced in rabbit	Sigma	C4731	Polyclonal	Rabbit	WB 1:10000
ERC1	Anti-ERC1 [ELKS-30] Against residues 21-40 of ERC1a	Abcam	ab50312	Monoclonal	Mouse	WB 1:1000
ERC1	Anti-ERC1	Sigma-Aldrich	HPA019513	Polyclonal	Rabbit	IF 1:150
FLAG	Monoclonal ANTI-FLAG^®^ M2, clone M2	Sigma-Aldrich	F1804	Monoclonal	Mouse	WB 1:1000 IF 1:500
GFP	GFP Polyclonal Antibody	Invitrogen	A11122	Polyclonal	Rabbit	WB 1:2000 IP 2 µg
GFP	Anti-GFP antibody	Abcam	ab13970	Polyclonal	Chicken	IF 1:1000
Lamins	Anti-Lamin A + Lamin B1 + Lamin C	Abcam	Ab108922	Monoclonal	Rabbit	WB 1:5000
Liprin-α1	Anti-liprin-α1 (A-5)	Santa Cruz Biotechnology	sc-376141	Monoclonal	Mouse	IP 0.5 µg IF 1:50
Liprin-α1	Anti-liprin-α1	Proteintech	14175-1-AP	Polyclonal	Rabbit	WB 1:500 IF 1:150
Paxillin	Purified Mouse Anti-Paxillin	BD Transduction Laboratories^™^	610052	Monoclonal	Mouse	WB 1:2000 IF 1:150 IP 2 µg
Paxillin	Paxillin antibody	GeneTex	GTX125891	Polyclonal	Rabbit	IF 1:200
Src	Clone 327 from S. Courtneidge	Monoclonal	Mouse	IF 1:50		
pSrc	Phospho-Src Family (Tyr416)	Cell Signaling Technology	#2101	Polyclonal	Rabbit	IF 1:100
Tubulin	Monoclonal anti-α-Tubulin	Sigma-Aldrich	T9026	Monoclonal	Mouse	WB 1:4000

*Secondary antibodies*				
*Antibody*	*Conjugation*	*Supplier*	*Cat. No*.	*Comment/Use*
Anti-rabbit IgG	HRP	Jackson	111-035-144	WB 1:5000
Anti-mouse IgG	HRP	Jackson	115-035-003	WB 1:5000
Anti-mouse IgG for IP	HRP	Abcam	ab131368	WB 1:3000
Anti-mouse	Alexa Fluor 488	Thermo Scientific	A21202	IF 1:200
Anti-mouse IgG1	Alexa Fluor 568	Thermo Scientific	A21124	IF 1:200
Anti-mouse	Alexa Fluor 546	Thermo Scientific	A10036	IF 1:200
Anti-mouse IgG1	Alexa Fluor 647	Thermo Scientific	A21240	IF 1:200
Anti-mouse	Alexa Fluor 647	Thermo Scientific	A31571	IF 1:200
Anti-rabbit	Alexa Fluor 488	Thermo Scientific	A11008	IF 1:200
Anti-rabbit	Alexa Fluor 488	Thermo Scientific	A21206	IF 1:200
Anti-rabbit	Alexa Fluor 568	Thermo Scientific	A10042	IF 1:200
Anti-rabbit	Alexa Fluor 647	Thermo Scientific	A31573	IF 1:200
Anti-chicken	Alexa Fluor 488	Thermo Scientific	A11039	IF 1:200

### Cell fractionation

To achieve the separation of nuclear and cytoplasmic proteins, the REAP protocol was employed with minor changes^49^. Adherent cells were washed twice with cold PBS, collected in PBS with the help of a scraper, and pelleted in a refrigerated centrifuge. Cells were resuspended in lysis buffer (0,1% NP-40, 0.5 mM PMSF, anti-proteases Complete 1×, and 1 mM DTT in PBS) and triturated ten times with a p1000 micropipette. A fraction of whole cell extract was saved, prior to proceed with a “pop-spin”. The supernatant (cytosolic fraction) was removed, and the pellet resuspended in lysis buffer (nuclear fraction). Total and nuclear fractions were sonicated (5” for 3 cycles). Samples buffer was added to each sample, prior to a 5′ incubation at 95 °C, and SDS-PAGE. Tubulin and Lamins were used as controls for cytosolic and nuclear fractions, respectively.

### Alkaline treatment (NaOH)

Cell lysates were subject to alkaline treatment (NaOH) or control treatment (pre-neutralized alkaline buffer), as described^23^. Accordingly, each sample was divided into two tubes: in the first one, NaOH was added to reach a final concentration of 0.2 M, while a neutral solution of NaOH/HCl (final concentration 0.2 M each) was added to the second. Treated samples were kept at room temperature for 5–10 min and finally neutralized with HCl (final concentration 0.2 M). Samples were boiled with protein sample buffer and analyzed by immunoblotting.

### Immunofluorescence and image analysis

Transfected cells were processed for immunofluorescence as described^50^. Briefly, cells were fixed for 10 min with 3% paraformaldehyde at room temperature, permeabilized with 0.1% Triton-X100 in PBS, incubated with primary antibodies, washed, incubated with secondary antibodies, and mounted with ProLong Gold antifade mounting solution (Thermo Fisher Scientific). Cells were observed with epifluorescence microscopes: Zeiss AxioImager M2m equipped with AxioCam color CCD camera, with Plan-Neofluar 40× lens (NA 0.75) and Plan-Apochromat 63× lens (NA 1.4). Confocal images were acquired at a Perkin Elmer UltraVIEW spinning disk confocal microscope with EM-CCD camera and Plan-Apochromat 63× lens (NA 1.4); or at a Leica TCS SP5 or TCS SP8 SMD FLIM laser scanning confocal microscope equipped with HC PLAPO CS2 63x lens (NA 1.4). For quantitative analysis of the projected cell area, transfected cells were randomly imaged at a wide field microscope (Zeiss Axio Observer.Z1 equipped with Hamamatsu 9100 - 02 EM CCD Camera). For evaluation of the subcellular localization of transfected and of endogenous proteins, confocal images were visually analyzed. For quantification, 2–4 independent experiments per condition were analyzed using Fiji^51^.

### Saponin-treatment and quantification of proteins in PMAPs

Saponin treatment before fixation and immunofluorescence was used to determine the capacity of B56γ-GFP to associate with PMAPs. Briefly, MDA-MB-231 cells were transfected with B56γ-GFP in combination with siRNAs (siCtr, siLip or siERC1) and seeded on fibronectin-coated (10 μg/ml) coverslips the next day. After overnight incubation, cells were washed with cold PBS once, treated with 0.05% saponin in CSB (115 mM potassium acetate, 25 mM HEPES pH 7.5, 2.5 mM MgCl_2_, 1 mM EGTA pH 7.5, 0.2 mM CaCl_2_, 12 mM glucose, 10 mM NAF, 1 mM NAV, 0.5 mM PMSF, anti-proteases 1× Complete^®^) for 5 min on ice, washed with cold CSB, and fixed with 3 % PFA at room temperature for 10 min.

Immunofluorescence was carried out as described above. Images were acquired using a Leica TCS SP5 confocal microscope with 63× lens (Leica microsystems). PMAPs were identified in virtue of the ERC1- or liprin-α1-positive signal. Once regions of interest were defined, the fluorescence intensity of each immunostained protein (B56γ-GFP, ERC1 and liprin-α1) was measured. To compensate for possible variations among experiments, the mean intensity of each protein on the control sample (cells cotransfected with B56γ-GFP and siCtr) was always considered equal to 1, and all measures expressed with respect to it. The B56γ-GFP fluorescence intensity within the nucleus was quantified to ensure comparable expression levels of the protein among samples.

### Functional analysis

For cell spreading, MDA-MB-231 or COS7 cells were transfected with the indicated plasmid and/or siRNA. After 1 day, cells were replated on fibronectin-coated glass coverslips (10 µg/ml, overnight at 4 °C), and fixed after 18 h (MDA-MD-231) or 1 h culture (COS7). After immunofluorescence, the projected cell area of the transfected cells was quantified by ImageJ software (NIH, Bethesda, MD).

For random migration, MDA-MB-231 cells were plated, transfected, replated and acquired as previously described^12^. Briefly, 50,000 transfected cells were seeded overnight on 2.5 mg/ml fibronectin-coated 6-well plate before time lapse with IncuCyte Live-Cell Imaging System equipped with 10× lens (Essen BioScience). Path, mean velocity, directionality and lamellipodia dynamics were evaluated during 5 h recording with ImageJ. Cells undergoing division and non-moving cells were ignored. The analysis of the frequency and persistence of lamellipodia was performed on frames from time-lapses for random migration assays according to a published protocol^13^.

For Matrigel^TM^ (BD Transduction) invasion, MDA-MB-231 cells transfected for 48 h with the indicated siRNAs were seeded on Matrigel^TM^–coated transwells (0.8 μm pores, Millipore) in DMEM 0.1% BSA (100,000 cells in 100 μl/transwell), with lower chambers filled with NIH 3T3-conditioned medium. Cells were fixed after 5 h culture. Cells transfected with siRNAs were fixed with MetOH and colored with Crystal Violet or DAPI for quantification. Invading cells at the bottom of the transwell membrane were counted (*n* = 4–6 transwells per experimental condition, from 2 to 3 experiments).

### Fluorescent-gelatin degradation assay

Gelatin degradation was detected as published^27,52^. Glass coverslips coated for 1 h at room T with 0.5 mg/ml poly-L-lysine (Sigma-Aldrich) were quenched 15 min at 4 °C with 0.5% glutaraldehyde in PBS, and then coated for 10 min at room T with Oregon–green–conjugated gelatin (Life Technologies) diluted 1:4 in 0.2% gelatin in PBS. Subsequently the coverslips were additionally coated with 10 μg/ml fibronectin in PBS for 1 h at 37 °C. Cells were plated on gelatin-coated coverslips for 5 h before fixation and immunostaining. Gelatin degradation was detected at a Zeiss Axio Observer.Z1 equipped with Hamamatsu 9100 - 02 EM CCD Camera and Plan-Apochromat 63x (NA 1.4) lens. The dark areas of gelatin degradation and the projected cell areas were quantified by ImageJ on thresholded images. Data were pooled from 2 to 3 independent experiments.

### Statistics and reproduciblity

Statistical analysis was performed using GraphPad Prism 9.0. All datasets were tested for normality using Shapiro–Wilk test. For datasets with normal distribution, the statistical significance was calculated using unpaired two-tailed Student’s *t* test or one-way ANOVA with Dunnett’s or Tukey’s post-hoc. For datasets with non-normal distribution, the statistical significance was calculated using Kruskal–Wallis test with Dunn’s post-hoc. Data are presented as mean ± SEM. All experiments including biochemical analyses were repeated at least twice. For all figures: ns > 0.05; * indicates *p* ≤ 0.05; ** indicates *p* ≤ 0.01; *** indicates *p* ≤ 0.001. Mean values are expressed ± SEM.

### Reporting summary

Further information on research design is available in the Nature Research Reporting Summary linked to this article.

## Supplementary information

Supplementary Information

Description of Additional Supplementary Files

Supplementary Movie 1

Supplementary Movie 2

Supplementary Movie 3

Reporting Summary

## Data Availability

All figures listed have associated raw data: microscopy and immunoblotting images, and data for graphs supporting the results presented in this study are available in the San Raffaele Open Research Data Repository (ORDR, https://ordr.hsr.it/research-data/) with the DOI: 10.17632/wvt7kgsjvx.1^53^. Other information is available from the corresponding author upon reasonable request. Unedited gels are shown in Supplementary Fig. 7 (from Fig. 1), Supplementary Fig. 8 (from Figs. 3, 4) and in Supplementary Fig. 9 (from Supplementary Figs. 1, 3–6).

## References

[1] Janssens V, Goris J (2001). Protein phosphatase 2A: a highly regulated family of serine/threonine phosphatases implicated in cell growth and signalling. Biochem. J..

[2] Lechward K, Awotunde OS, Swiatek W, Muszynska G (2001). Protein phosphatase 2A: variety of forms and diversity of functions. Acta Biochim. Pol..

[3] Virshup DM (2000). Protein phosphatase 2A: a panoply of enzymes. Curr. Opin. Cell Biol..

[4] Larsen M, Tremblay ML, Yamada KM (2003). Phosphatases in cell-matrix adhesion and migration. Nat. Rev. Mol. Cell Biol..

[5] Shi Y (2009). Serine/threonine phosphatases: mechanism through structure. Cell.

[6] Perrotti D, Neviani P (2013). Protein phosphatase 2A: a target for anticancer therapy. Lancet Oncol..

[7] Hertz EPT (2016). A Conserved Motif Provides Binding Specificity to the PP2A-B56 Phosphatase. Mol. Cell.

[8] Van Roey K (2014). Short linear motifs: ubiquitous and functionally diverse protein interaction modules directing cell regulation. Chem. Rev..

[9] Pehkonen H, de Curtis I, Monni O (2021). Liprins in oncogenic signaling and cancer cell adhesion. Oncogene.

[10] Astro V, de Curtis I (2015). Plasma membrane-associated platforms: dynamic scaffolds that organize membrane-associated events. Sci. Signal..

[11] Ramella M, Ribolla LM, de Curtis I (2022). Liquid-Liquid Phase Separation at the Plasma Membrane-Cytosol Interface: Common Players in Adhesion, Motility, and Synaptic Function. J. Mol. Biol..

[12] Astro V, Asperti C, Cangi MG, Doglioni C, de Curtis I (2011). Liprin-α1 regulates breast cancer cell invasion by affecting cell motility, invadopodia and extracellular matrix degradation. Oncogene.

[13] Astro V, Chiaretti S, Magistrati E, Fivaz M, de Curtis I (2014). Liprin-α1, ERC1 and LL5 define polarized and dynamic structures that are implicated in cell migration. J. Cell Sci..

[14] Astro V (2016). liprin-alpha1 and ERC1 control cell edge dynamics by promoting focal adhesion turnover. Sci. Rep..

[15] Arroyo JD, Lee GM, Hahn WC (2008). Liprin alpha1 interacts with PP2A B56gamma. Cell Cycle.

[16] Li L (2014). Drosophila Syd-1, liprin-α, and protein phosphatase 2A B’ subunit Wrd function in a linear pathway to prevent ectopic accumulation of synaptic materials in distal axons. J. Neurosci..

[17] Wu CG (2017). PP2A-B’ holoenzyme substrate recognition, regulation and role in cytokinesis. Cell Disco..

[18] Davey NE, Cyert MS, Moses AM (2015). Short linear motifs - ex nihilo evolution of protein regulation. Cell Commun. Signal..

[19] Tolstykh T, Lee J, Vafai S, Stock JB (2000). Carboxyl methylation regulates phosphoprotein phosphatase 2A by controlling the association of regulatory B subunits. EMBO J..

[20] Wu J (2000). Carboxyl methylation of the phosphoprotein phosphatase 2A catalytic subunit promotes its functional association with regulatory subunits in vivo. EMBO J..

[21] Yu XX (2001). Methylation of the protein phosphatase 2A catalytic subunit is essential for association of Balpha regulatory subunit but not SG2NA, striatin, or polyomavirus middle tumor antigen. Mol. Biol. Cell.

[22] Janssens V, Longin S, Goris J (2008). PP2A holoenzyme assembly: in cauda venenum (the sting is in the tail). Trends Biochem. Sci..

[23] Frohner, I.E., Mudrak, I., Kronlachner, S., Schuchner, S. & Ogris, E. Antibodies recognizing the C terminus of PP2A catalytic subunit are unsuitable for evaluating PP2A activity and holoenzyme composition. *Sci. Signal.* **13**, eaax6490, 10.1126/scisignal.aax6490 (2020). 10.1126/scisignal.aax6490 31992581

[24] McCright B, Rivers AM, Audlin S, Virshup DM (1996). The B56 family of protein phosphatase 2A (PP2A) regulatory subunits encodes differentiation-induced phosphoproteins that target PP2A to both nucleus and cytoplasm. J. Biol. Chem..

[25] Kim KY (2009). Adiponectin-activated AMPK stimulates dephosphorylation of AKT through protein phosphatase 2A activation. Cancer Res..

[26] de Curtis I, Malanchini B (1997). Integrin-mediated tyrosine phosphorylation and redistribution of paxillin during neuronal adhesion. Exp. Cell Res..

[27] Sala K, Raimondi A, Tonoli D, Tacchetti C, de Curtis I (2018). Identification of a membrane-less compartment regulating invadosome function and motility. Sci. Rep..

[28] Chiaretti S, Astro V, Chiricozzi E, de Curtis I (2016). Effects of the scaffold proteins liprin-α1, β1 and β2 on invasion by breast cancer cells. Biol. Cell.

[29] Foley EA, Maldonado M, Kapoor TM (2011). Formation of stable attachments between kinetochores and microtubules depends on the B56-PP2A phosphatase. Nat. Cell Biol..

[30] Porter IM, Schleicher K, Porter M, Swedlow JR (2013). Bod1 regulates protein phosphatase 2A at mitotic kinetochores. Nat. Commun..

[31] Eddy RJ, Weidmann MD, Sharma VP, Condeelis JS (2017). Tumor Cell Invadopodia: Invasive Protrusions that Orchestrate Metastasis. Trends Cell Biol..

[32] Asperti C, Astro V, Totaro A, Paris S, de Curtis I (2009). Liprin-alpha1 promotes cell spreading on the extracellular matrix by affecting the distribution of activated integrins. J. Cell Sci..

[33] Young MR, Kolesiak K, Meisinger J (2002). Protein phosphatase-2A regulates endothelial cell motility and both the phosphorylation and the stability of focal adhesion complexes. Int. J. Cancer.

[34] Young MR, Liu SW, Meisinger J (2003). Protein phosphatase-2A restricts migration of Lewis lung carcinoma cells by modulating the phosphorylation of focal adhesion proteins. Int. J. Cancer.

[35] Ripamonti M, Wehrle-Haller B, de Curtis I (2022). Paxillin: A Hub for Mechano-Transduction from the β3 Integrin-Talin-Kindlin Axis. Front. Cell Dev. Biol..

[36] Bouchet BP (2016). Talin-KANK1 interaction controls the recruitment of cortical microtubule stabilizing complexes to focal adhesions. Elife.

[37] Sun Z (2016). Kank2 activates talin, reduces force transduction across integrins and induces central adhesion formation. Nat. Cell Biol..

[38] Ito A (2000). A truncated isoform of the PP2A B56 subunit promotes cell motility through paxillin phosphorylation. EMBO J..

[39] Jackson JL, Young MR (2002). Protein phosphatase-2A modulates the serine and tyrosine phosphorylation of paxillin in Lewis lung carcinoma tumor variants. Clin. Exp. Metastasis.

[40] Chen W (2004). Identification of specific PP2A complexes involved in human cell transformation. Cancer Cell.

[41] Cho US, Xu W (2007). Crystal structure of a protein phosphatase 2A heterotrimeric holoenzyme. Nature.

[42] Jackson JB, Pallas DC (2012). Circumventing cellular control of PP2A by methylation promotes transformation in an Akt-dependent manner. Neoplasia.

[43] Ambjorn SM (2021). A complex of BRCA2 and PP2A-B56 is required for DNA repair by homologous recombination. Nat. Commun..

[44] Sala K (2019). The ERC1 scaffold protein implicated in cell motility drives the assembly of a liquid phase. Sci. Rep..

[45] de Curtis I (2021). Biomolecular Condensates at the Front: Cell Migration Meets Phase Separation. Trends Cell Biol..

[46] Gigena MS, Ito A, Nojima H, Rogers TB (2005). A B56 regulatory subunit of protein phosphatase 2A localizes to nuclear speckles in cardiomyocytes. Am. J. Physiol. Heart Circ. Physiol..

[47] Meng F (2021). Induced phase separation of mutant NF2 imprisons the cGAS-STING machinery to abrogate antitumor immunity. Mol. Cell..

[48] Erpel T, Superti-Furga G, Courtneidge SA (1995). Mutational analysis of the Src SH3 domain: the same residues of the ligand binding surface are important for intra- and intermolecular interactions. EMBO J..

[49] Suzuki K, Bose P, Leong-Quong RY, Fujita DJ, Riabowol K (2010). REAP: a two minute cell fractionation method. BMC Res. Notes.

[50] Totaro A, Paris S, Asperti C, de Curtis I (2007). Identification of an intramolecular interaction important for the regulation of GIT1 functions. Mol. Biol. Cell.

[51] Schindelin J (2012). Fiji: an open-source platform for biological-image analysis. Nat. Methods.

[52] Artym VV, Zhang Y, Seillier-Moiseiwitsch F, Yamada KM, Mueller SC (2006). Dynamic interactions of cortactin and membrane type 1 matrix metalloproteinase at invadopodia: defining the stages of invadopodia formation and function. Cancer Res..

[53] de Curtis I (2022). Data repository for Ripamonti et al. Commun. Biol..

