# Supplementary material for: A functional interaction between liprin-α1 and B56γ regulatory subunit of protein phosphatase 2A supports tumor cell motility
Source: Commun Biol. 2022 Sep 28;5:1025. doi: 10.1038/s42003-022-03989-3 (PMC9519923; doi:10.1038/s42003-022-03989-3)
Supplement: Supplementary file 1 — Supplementary Information [file 42003_2022_3989_MOESM1_ESM.pdf]

A functional interaction between liprin-α1 and B56γ regulatory subunit of protein phosphatase 2A supports tumor cell motility

Marta Ripamonti[1#], Andrea Lamarca[1#], Norman E. Davey[2], Diletta Tonoli[1], Sara Surini[1], Ivan de Curtis[1]

[1] San Raffaele Scientific Institute and Università Vita-Salute San Raffaele, Milano, Italy

[2] Division of Cancer Biology, The Institute of Cancer Research, 237 Fulham Road, London SW3 6JB, UK.

[#] These authors contributed equally to this study.

The authors have no competing interests.

Corresponding author:

Ivan de Curtis

Cell Adhesion Unit, Division of Neuroscience

San Raffaele Scientific Institute and Università Vita-Salute San Raffaele,

Via Olgettina 58, 20132 Milano, Italy

Tel: +39 02 2643 4828; email: decurtis.ivan@hsr.it

Supplementary Figure 1. Expression of the liprin-α1/B56γ/PP2A-C complex in different breast cancer cell lines.

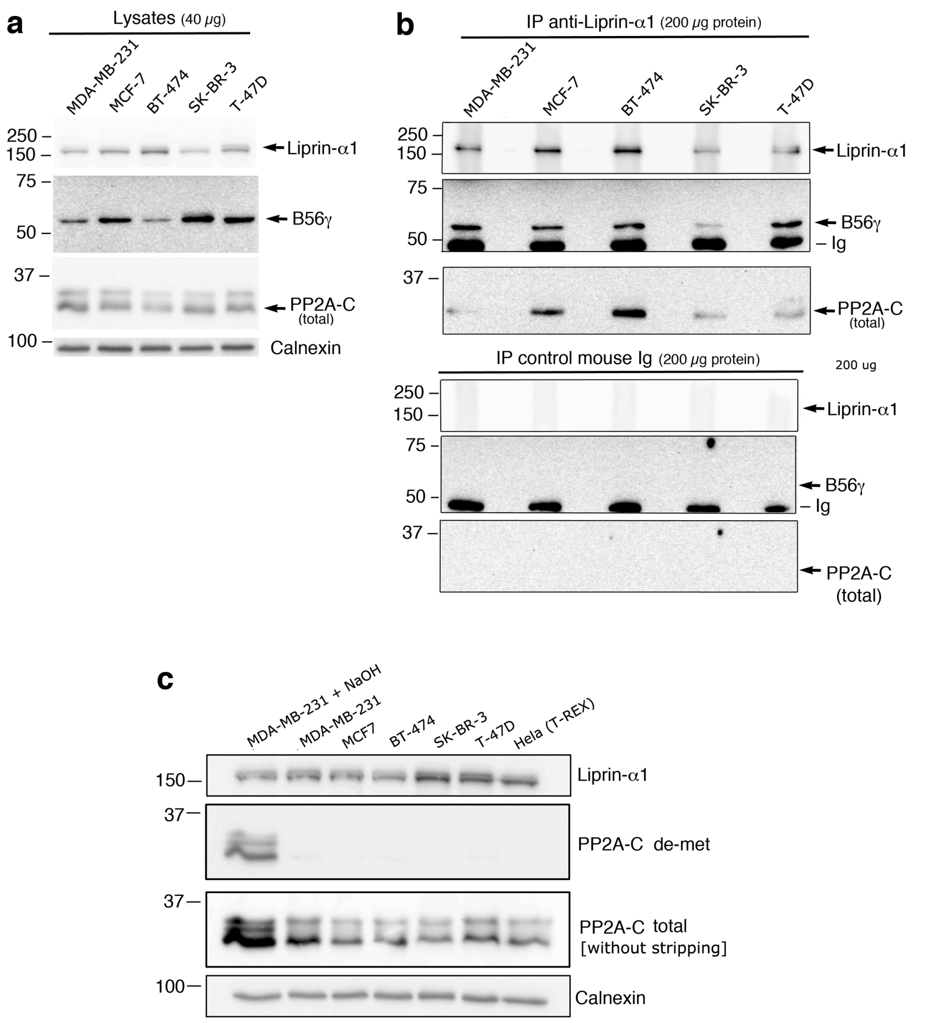

a Immunoblotting on lysates from indicated breast cancer cell lines. **b** Immunoprecipitation of endogenous liprin-α1 to detect the endogenous liprin-α1/B56γ/PP2A-C complexes. **c** The endogenous catalytic PP2A-C subunit in different breast cancer cell lines is methylated. Filters with cell lysates were incubated with Abs for the indicated antigens. The second filter from the top was incubated with an Ab specific for the demethylated form of PP2A-C; the first lane on the left has been loaded with an aliquot of lysate treated with NaOH to demethylate PP2A-C (positive control). The same filter was then re-blotted with an Ab recognizing both methylated and demethylated PP2A-C (PP2A-C total, third blot from the top).

Supplementary Figure 2. Subcellular localization or wildtype and mutant B56γ and liprin-α1 in migrating MDA-MB-231 cells.

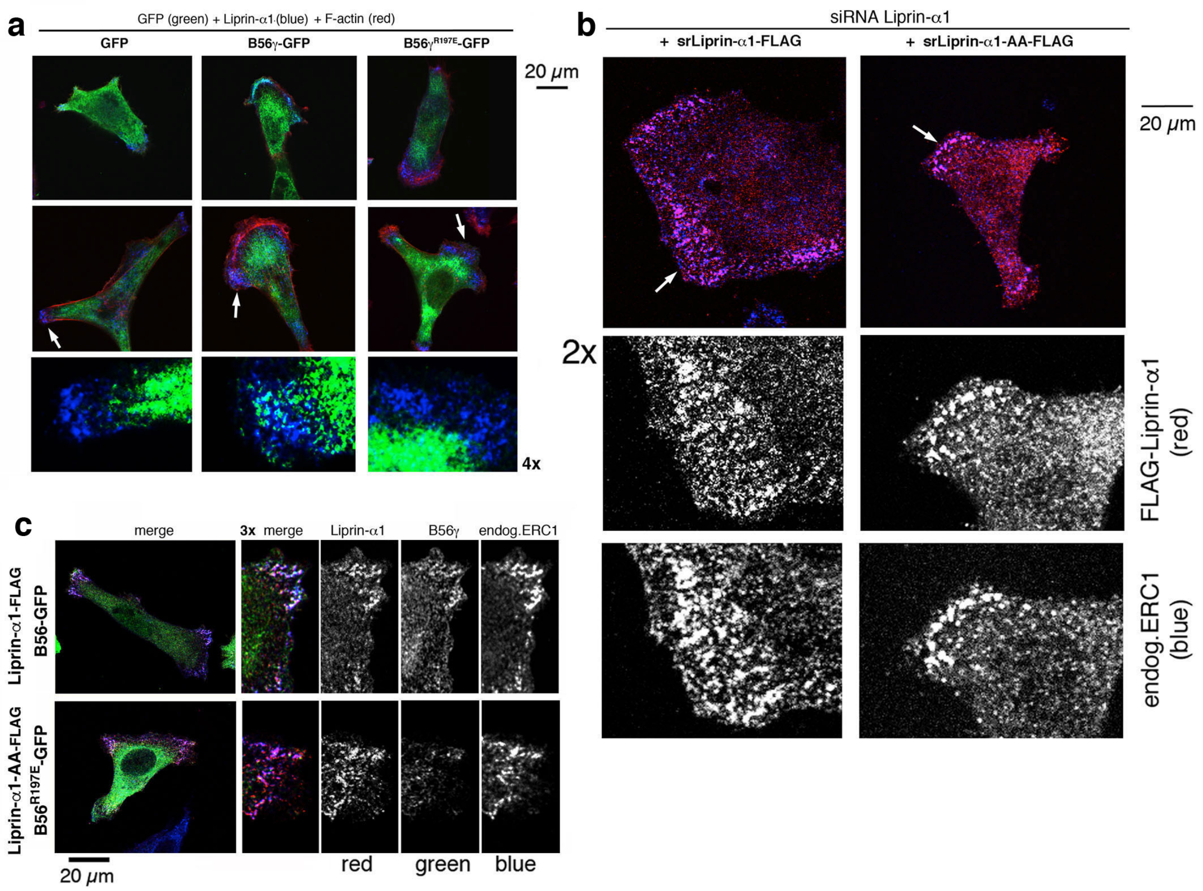

a Colocalization of B56γ-GFP with endogenous liprin-α1 at PMAPs in migrating MDA-MB-231 cells plated on 2.5 μg/ml fibronectin. **b** Both wildtype (left) and SLiM mutant liprin-α1 (right) localize at PMAPs. **C** B56γ-GFP colocalizes with liprin-α1-FLAG and endogenous ERC1 at PMAPs in migrating MDA-MB-231 cells.

Supplementary Figure 3. Liprin-α1 silencing affects the localization of B56γ at PMAPs.

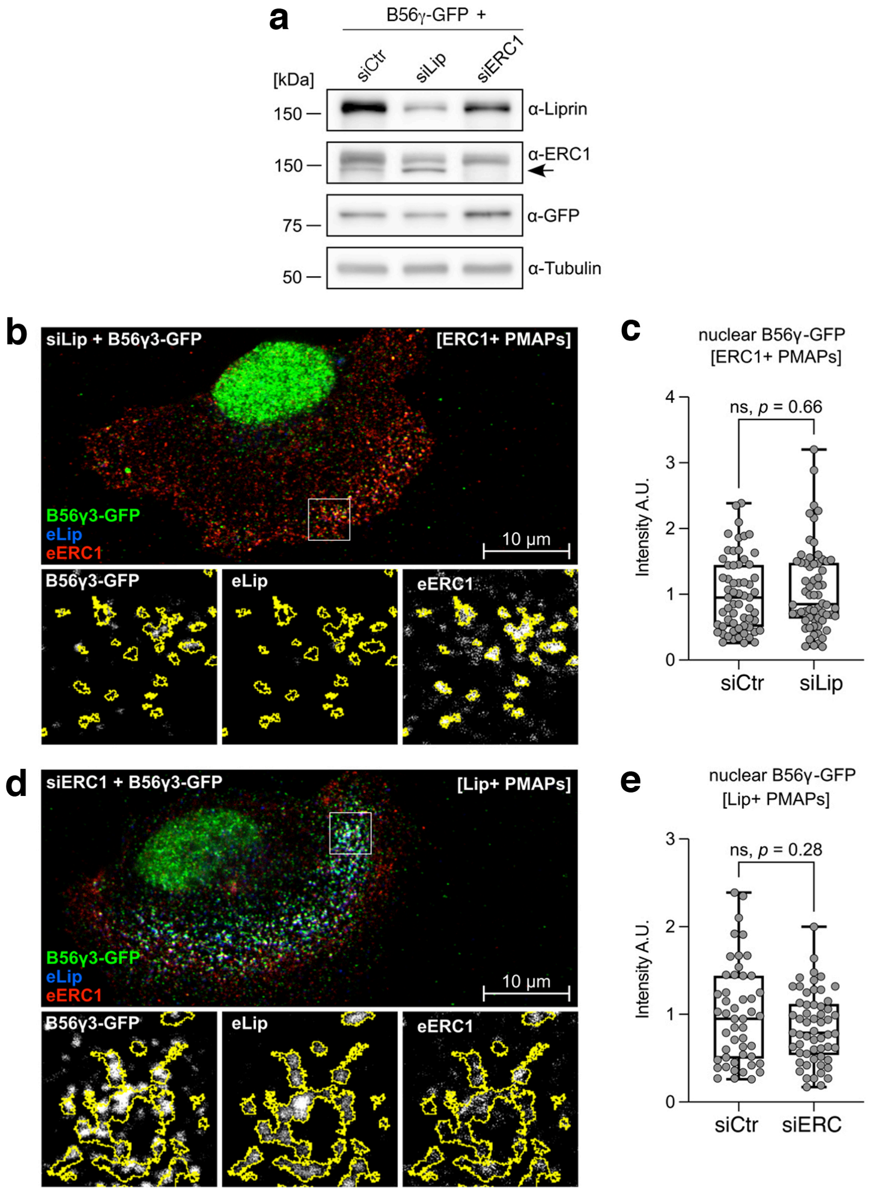

a Western blot of MDA-MB-231 cells cotransfected with B56γ-GFP and control (siCtr), liprin-α1 (siLip), or ERC1 (siERC1) siRNA. Immunoblotting for liprin-α1 and ERC1 (top filter reblotted for ERC1). **b** Quantification of signal for B56γ-GFP in ERC1-positive PMAPs: ROIs (yellow) were defined according to the signal of ERC1, and subsequently applied to B56γ-GFP and Liprin-α1 channels. **c** Quantification of nuclear signal of B56γ-GFP. **d** Quantification of B56γ-GFP in liprin-α1-positive PMAPs: ROIs (yellow) were defined for the signal of ERC1, and subsequently applied to B56γ-GFP and liprin-α1 channels. **E** Quantification of B56γ-GFP nuclear signal.

Supplementary Figure 4. Silencing of B56γ.

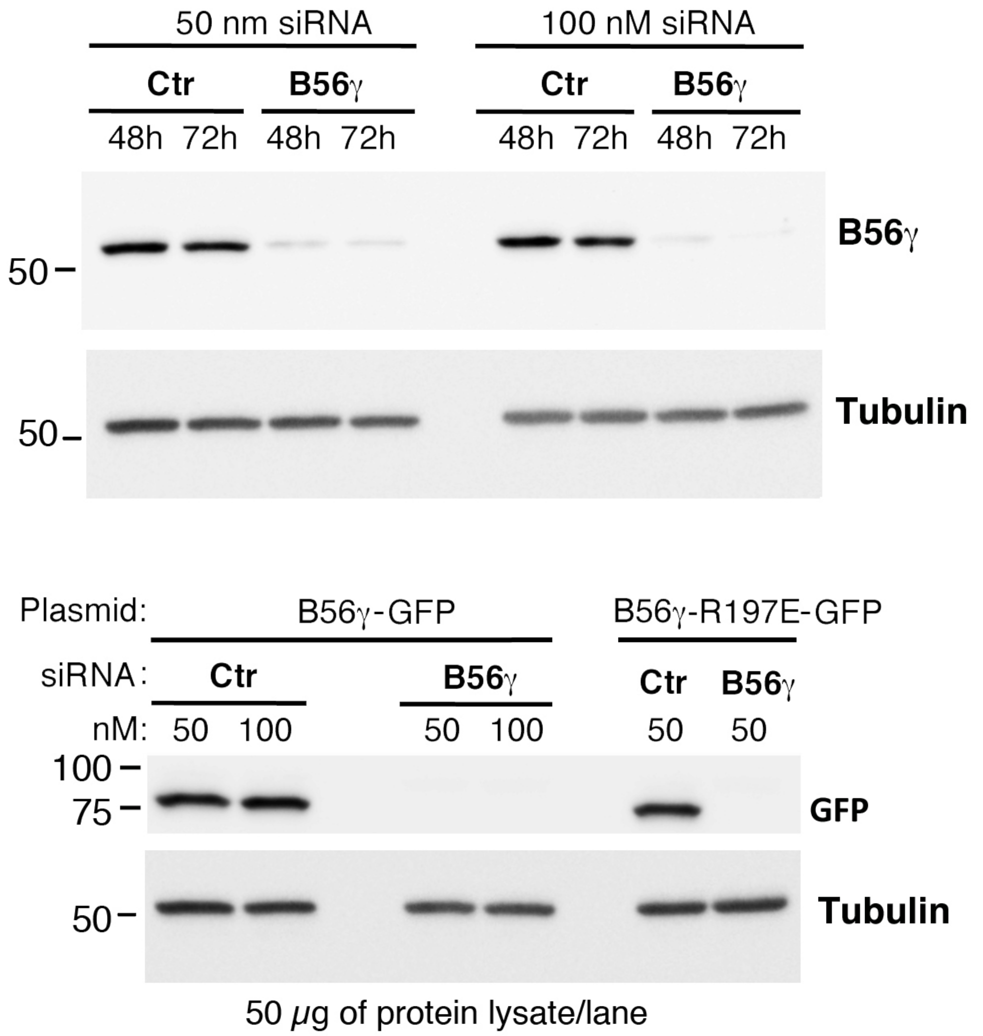

Silencing of endogenous (top) and overexpressed (bottom) B56γ in MDA-MB-231 cells transfected with the indicated siRNAs and plasmids.

Supplementary Figure 5. Localization of B56γ at PMAPs near invadosomes.

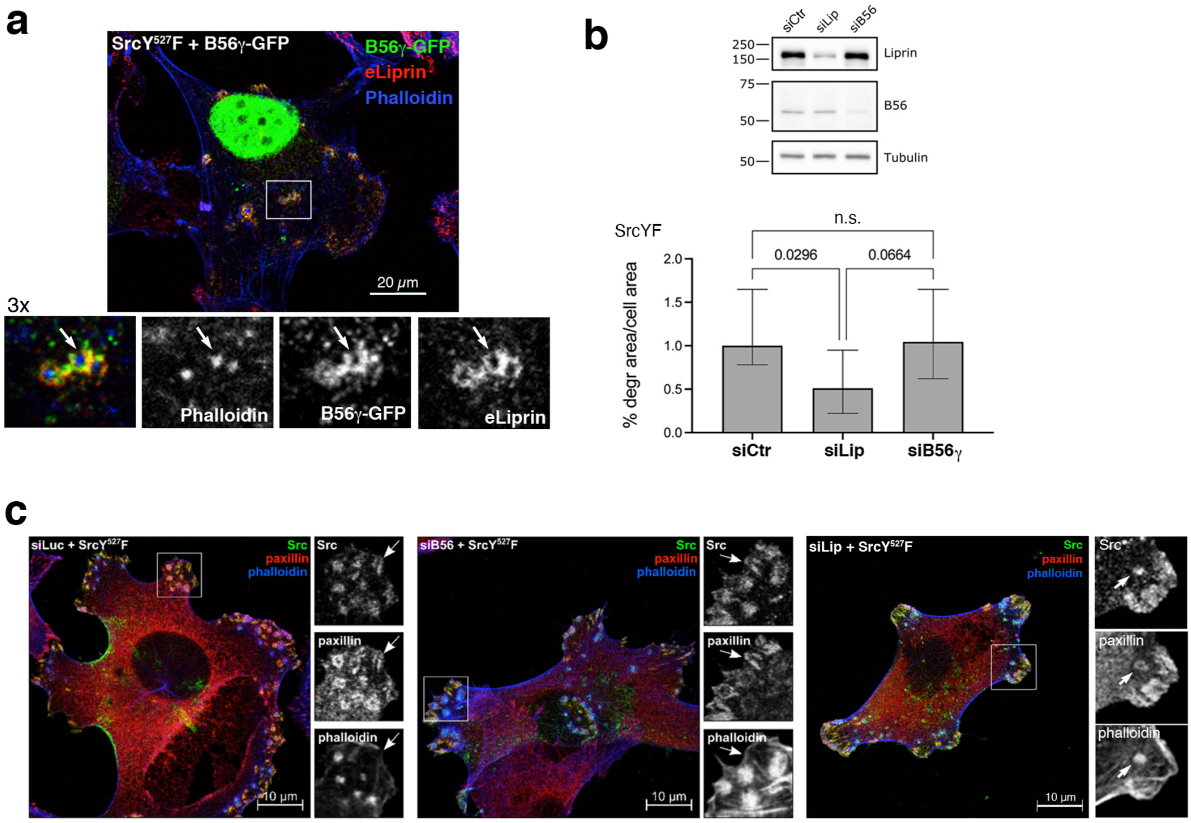

a Cotransfection of B56γ-GFP and constitutive active Src (SrcY^{527}F) showed the colocalization of B56γ-PP2A with liprin-α1 PMAPs near invadosomes. **b** Representative western blot of MDA-MB-231 cells cotransfected with constitutive active Src (SrcY^{527}F) and control (siCtr), liprin-α1 (siLip), or B56γ (siB56) siRNA. Graphs: quantification of the area of ECM degradation per cell (left) and of ECM degradation/cell area (right) (left graph, medians + 95% CI; n = 201-217 cells from 3 experiments; Kruskal-Wllis test and Dunn's correction for multiple comparisons; right graph, means + SEM). **c** Confocal images of SrcY^{527}F-transfected MDA-MB-231 cells with control, B56γ, or liprin-α1 siRNA. Paxillin accumulates at adhesions and around Src- and phalloidin-positive invadosomes. Merge images: Src (green), paxillin (red), phalloidin (blue).

Supplementary Figure 6. B56γ does not interact with paxillin.

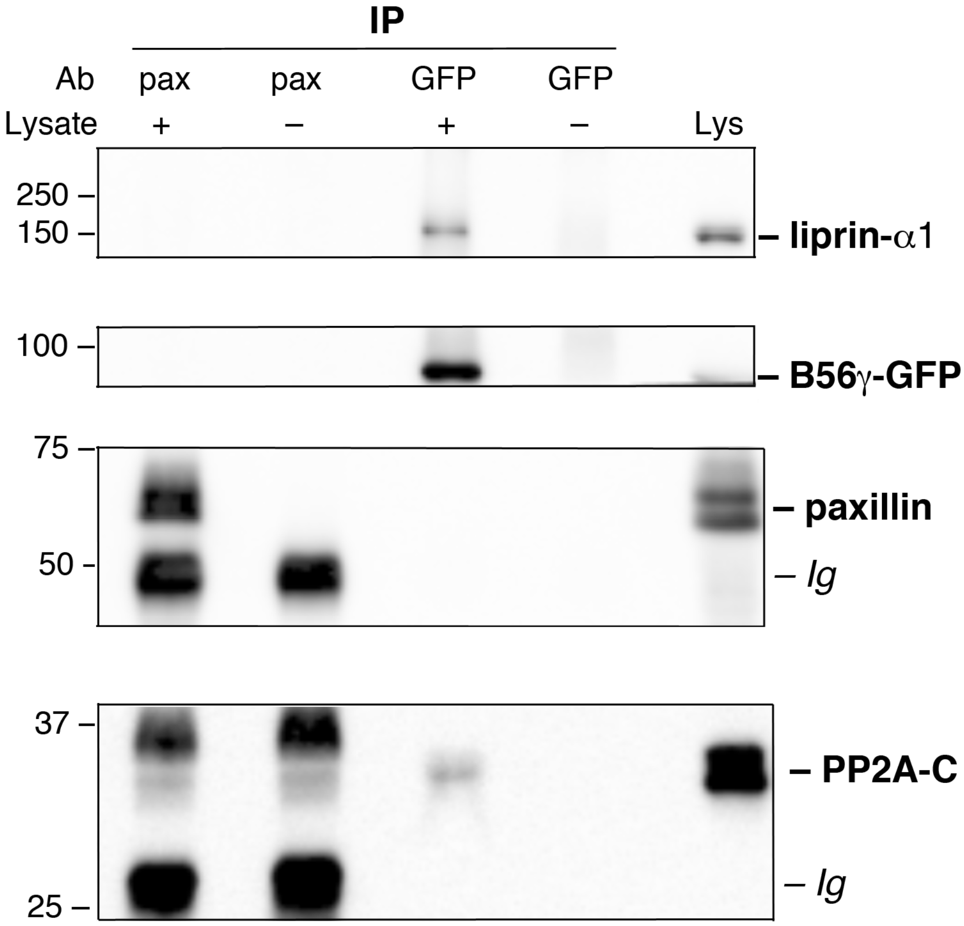

Immunoprecipitation with anti-paxillin (pax) or anti-GFP (GFP-trap) Abs from lysates of MDA-MB-231 cells transfected with B56γ-GFP. Filters were cut and incubated with the indicated Abs.

Supplementary Figure 7. Full, uncropped blot images of panels presented in Figure 1.

Blots Figure 1

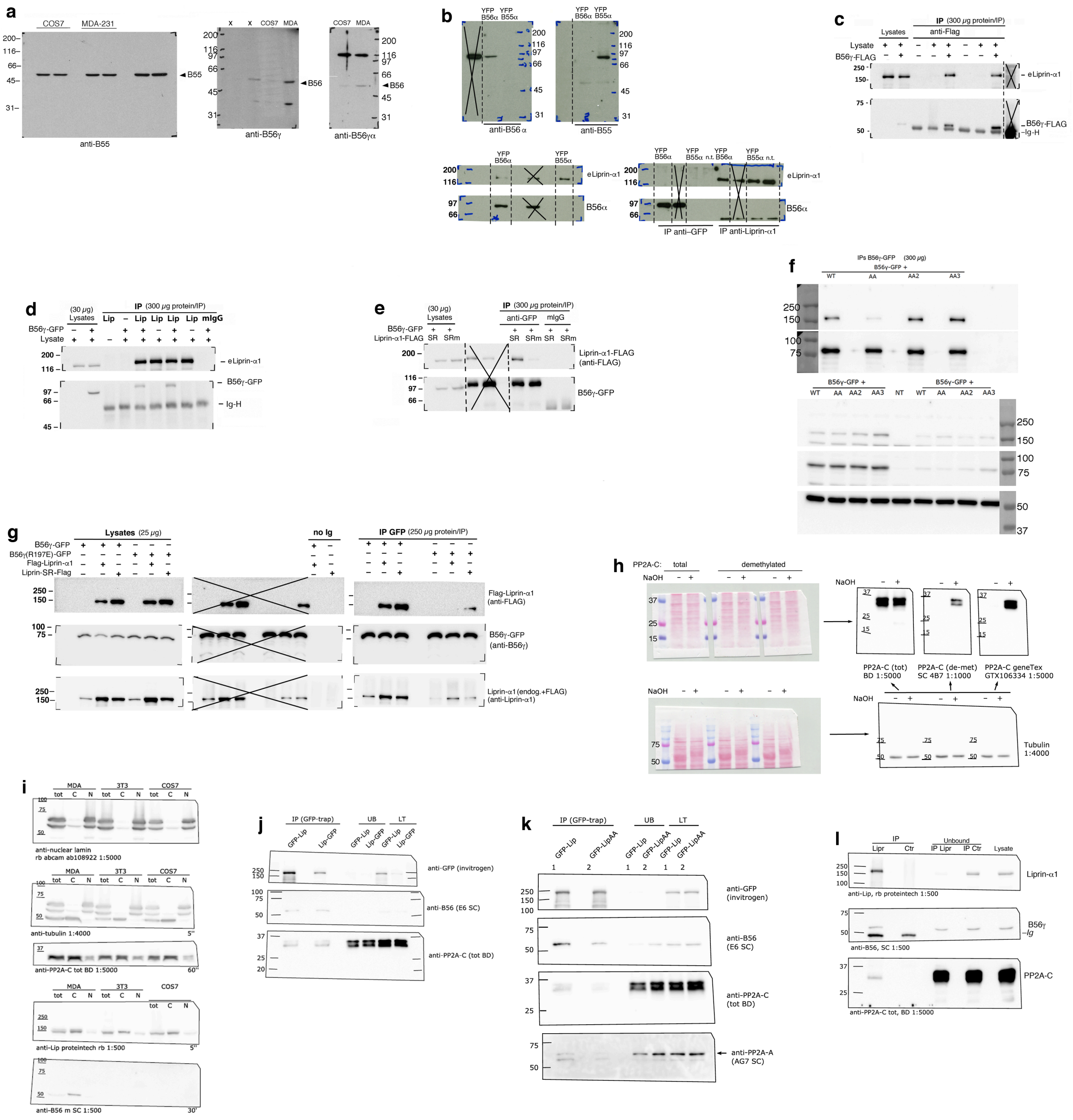

Unedited images of the blots shown in the indicated panels of **Figures 1**. The description of the experimental conditions are described in the legend of **Figure 1**.

Supplementary Figure 8. Full, uncropped blot images of panels presented in Figures 3 and 4.

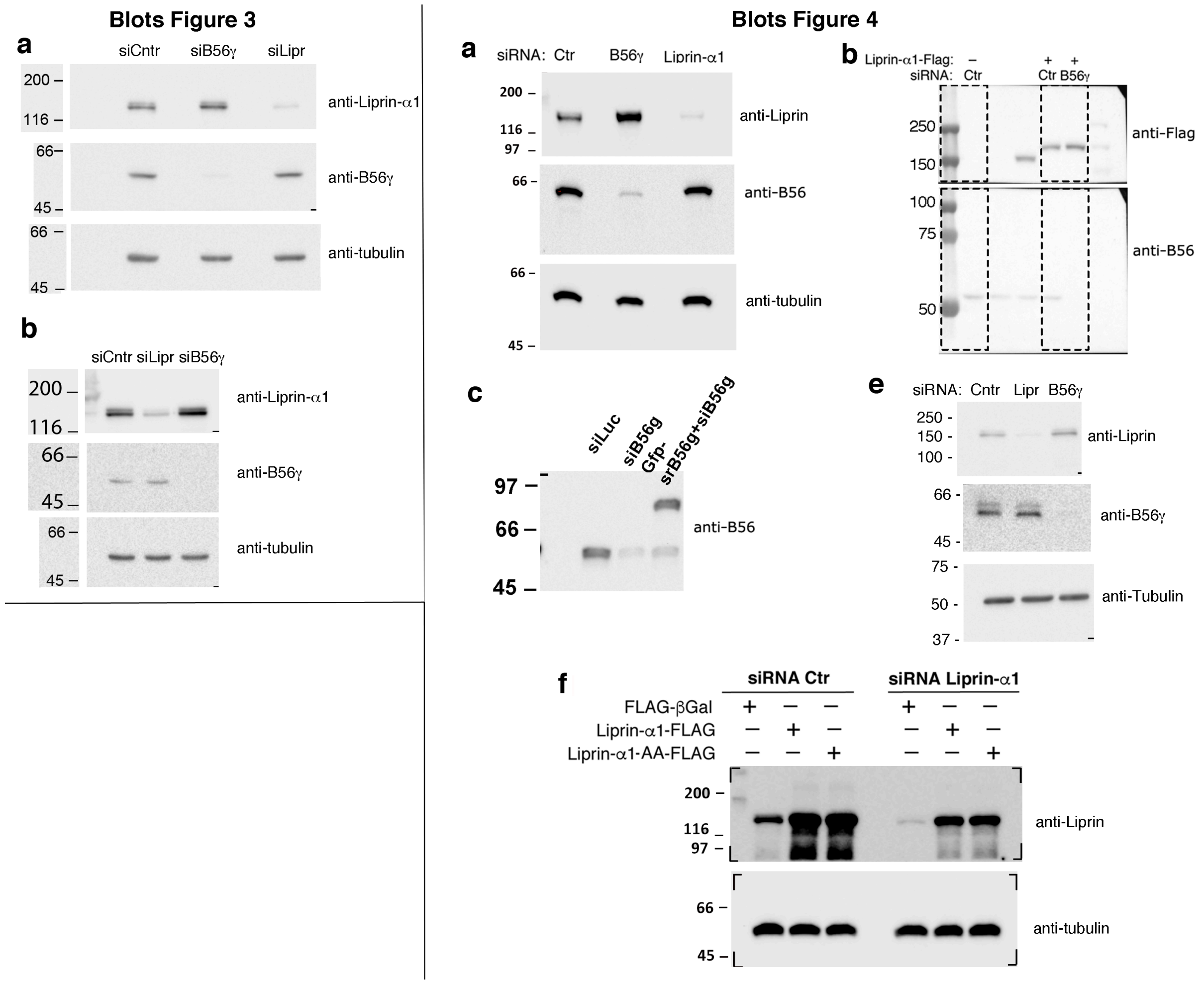

Unedited images of the blots shown in the indicated panels of **Figures 3** and **4**. The description of the experimental conditions are described in the legends of the respective figures.

Supplementary Figure 9. Full, uncropped blot images of panels presented in Supplementary Figures.

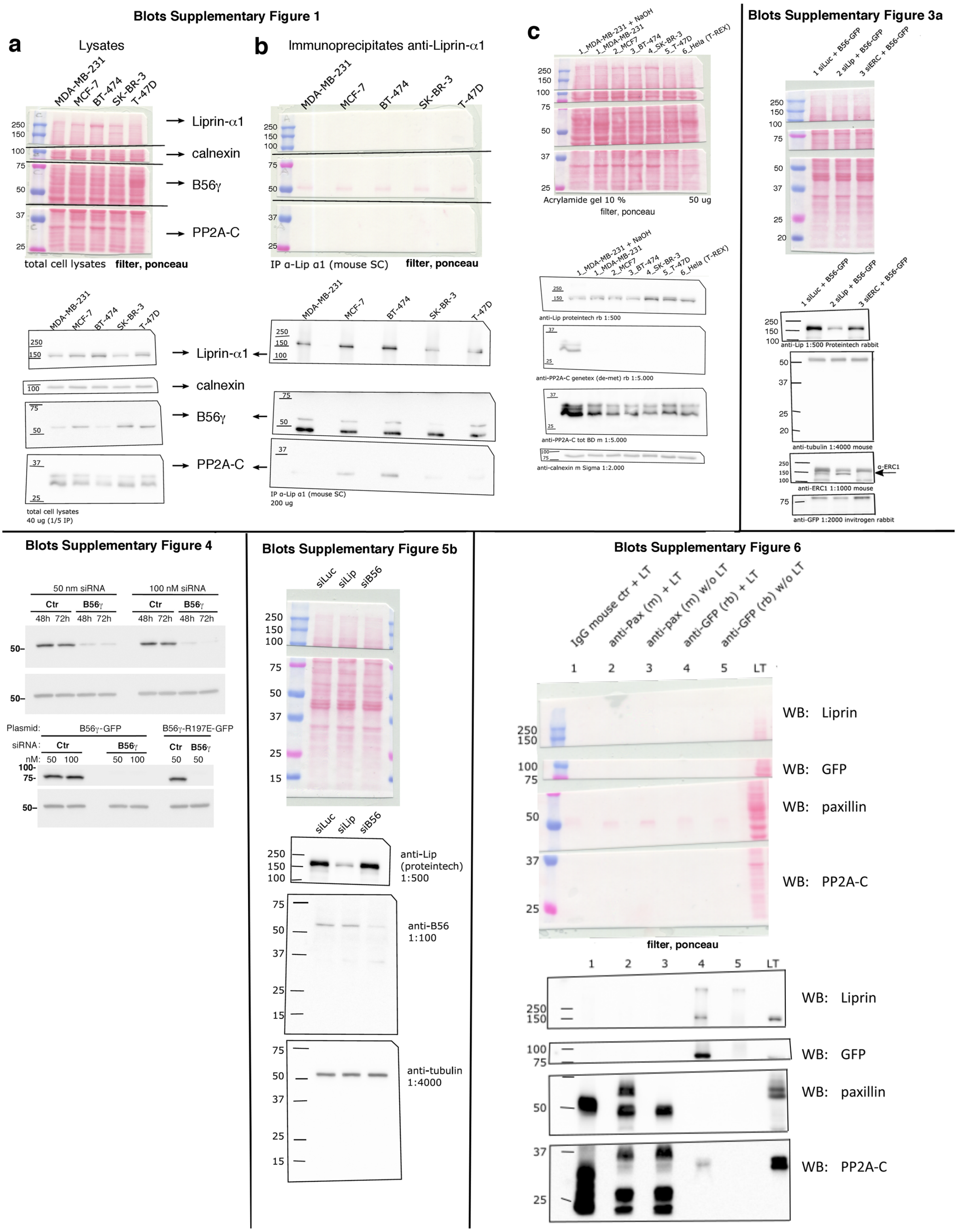

Unedited images of the blots shown in the indicated panels of **Supplementary Figures 1, 3, 4, 5, 6**. The description of the experimental conditions are described in the legends of the respective Supplementary Figures.
