# Supplementary material for: A functional interaction between liprin-α1 and B56γ regulatory subunit of protein phosphatase 2A supports tumor cell motility
Source: Commun Biol. 2022 Sep 28;5:1025. doi: 10.1038/s42003-022-03989-3 (PMC9519923; doi:10.1038/s42003-022-03989-3)
Supplement: Supplementary file 2 — Description of Additional Supplementary Files [file 42003_2022_3989_MOESM2_ESM.pdf]

# Description of Additional Supplementary Files

**File name: Supplementary Movie 1**

**Description**: Migration of MDA-231 cells transfected with siRNA for B56g. MDA-MB-231 cells cotransfected with siRNA for B56g and GFP were replated on wells coated 2.5 mg/ml fibronectin and recorded for 5 h.

**File name: Supplementary Movie 2**

**Description:** Migration of MDA-231 cells transfected with siRNA for Liprina1. MDA-MB-231 cells cotransfected with siRNA for Liprin-a1 and GFP were replated on wells coated 2.5 mg/ml fibronectin and recorded for 5 h.

**File name: Supplementary Movie 3**

**Description:** Migration of MDA-231 cells transfected with control siRNA. MDA-MB-231 cells cotransfected with control siRNA and GFP were replated on wells coated 2.5 mg/ml fibronectin and recorded for 5 h.

.
