# Supplementary material for: A functional interaction between liprin-α1 and B56γ regulatory subunit of protein phosphatase 2A supports tumor cell motility
Source: Commun Biol. 2022 Sep 28;5:1025. doi: 10.1038/s42003-022-03989-3 (PMC9519923; doi:10.1038/s42003-022-03989-3)
Supplement: Supplementary file 6 — Reporting Summary [file 42003_2022_3989_MOESM6_ESM.pdf]

nature portfolio

Corresponding author(s): Ivan de Curtis

Last updated by author(s): Ivan de Curtis

Reporting Summary

Nature Portfolio wishes to improve the reproducibility of the work that we publish. This form provides structure for consistency and transparency in reporting. For further information on Nature Portfolio policies, see our Editorial Policies and the Editorial Policy Checklist.

Statistics

For all statistical analyses, confirm that the following items are present in the figure legend, table legend, main text, or Methods section.

n/a	Confirmed	
☐	☒	The exact sample size (*n*) for each experimental group/condition, given as a discrete number and unit of measurement
☐	☒	A statement on whether measurements were taken from distinct samples or whether the same sample was measured repeatedly
☐	☒	The statistical test(s) used AND whether they are one- or two-sided<br>*Only common tests should be described solely by name; describe more complex techniques in the Methods section.*
☐	☒	A description of all covariates tested
☐	☒	A description of any assumptions or corrections, such as tests of normality and adjustment for multiple comparisons
☐	☒	A full description of the statistical parameters including central tendency (e.g. means) or other basic estimates (e.g. regression coefficient) AND variation (e.g. standard deviation) or associated estimates of uncertainty (e.g. confidence intervals)
☐	☒	For null hypothesis testing, the test statistic (e.g. *F*, *t*, *r*) with confidence intervals, effect sizes, degrees of freedom and *P* value noted<br>*Give P values as exact values whenever suitable.*
☒	☐	For Bayesian analysis, information on the choice of priors and Markov chain Monte Carlo settings
☒	☐	For hierarchical and complex designs, identification of the appropriate level for tests and full reporting of outcomes
☒	☐	Estimates of effect sizes (e.g. Cohen's *d*, Pearson's *r*), indicating how they were calculated

Our web collection on statistics for biologists contains articles on many of the points above.

Software and code

Policy information about availability of computer code

Data collection	-
Data analysis	-

For manuscripts utilizing custom algorithms or software that are central to the research but not yet described in published literature, software must be made available to editors and reviewers. We strongly encourage code deposition in a community repository (e.g. GitHub). See the Nature Portfolio guidelines for submitting code & software for further information.

Data

Policy information about availability of data

All manuscripts must include a data availability statement. This statement should provide the following information, where applicable:

- Accession codes, unique identifiers, or web links for publicly available datasets
- A description of any restrictions on data availability
- For clinical datasets or third party data, please ensure that the statement adheres to our policy

All figures listed have associated raw data: microscopy and immunoblotting images, and data for graphs supporting the results presented in this study are available in the San Raffaele Open Research Data Repository (ORDR, https://ordr.hsr.it/research-data/) with the DOI: 10.17632/wvt7kgsjvx.1

Human research participants

Policy information about studies involving human research participants and Sex and Gender in Research.

Reporting on sex and gender	-
Population characteristics	-
Recruitment	-
Ethics oversight	-

Note that full information on the approval of the study protocol must also be provided in the manuscript.

Field-specific reporting

Please select the one below that is the best fit for your research. If you are not sure, read the appropriate sections before making your selection.

☒ Life sciences ☐ Behavioural & social sciences ☐ Ecological, evolutionary & environmental sciences

For a reference copy of the document with all sections, see nature.com/documents/nr-reporting-summary-flat.pdf

Life sciences study design

All studies must disclose on these points even when the disclosure is negative.

Sample size	Sample sizes were based on previous experience with similar analysis, to achieve statistically significant differences.
Data exclusions	Generally no data were excluded. If data were excluded, this was specified in the manuscript.
Replication	Data were confirmed by replicating experiments successfully.
Randomization	Images from different experimental conditions were collected randomly, starting from one side of each coverslip (for immunofluorescence) until the established number of images was reached.
Blinding	Blinding was not relevant; images were collected randomly and quantitative analysis was based on objective parameters.

Reporting for specific materials, systems and methods

We require information from authors about some types of materials, experimental systems and methods used in many studies. Here, indicate whether each material, system or method listed is relevant to your study. If you are not sure if a list item applies to your research, read the appropriate section before selecting a response.

Materials & experimental systems

n/a	Involved in the study	
☐	☒	Antibodies
☐	☒	Eukaryotic cell lines
☒	☐	Palaeontology and archaeology
☒	☐	Animals and other organisms
☒	☐	Clinical data
☒	☐	Dual use research of concern

Methods

n/a	Involved in the study	
☒	☐	ChIP-seq
☒	☐	Flow cytometry
☒	☐	MRI-based neuroimaging

Antibodies

Antibodies used

PP2A-C Purified anti-PP2A catalytic α, clone 46 BD Transduction Laboratories™ 610556 Monoclonal Mouse
Recognizes methylated and and non-methylated form
WB 1:5000
PP2A-C Anti-demethylated-PP2A-C,
Clone 4B7 Santa Cruz Biotechnology sc-13601 Monoclonal Mouse Specific for non-methylated form
WB 1:1000
PP2A-C Anti-PP2A-Cα/β, Clone 1D6 Santa Cruz Biotechnology sc-13601 Monoclonal Mouse

Preferential recognition of non- methylated form
WB 1:1000
PP2A-C Anti-PP2A alpha GeneTex GTX106334 Polyclonal Rabbit Specific for non-methylated form
WB 1:5000
B55α Anti-B55α
Clone 2G9 Cell Signaling 5689 Monoclonal Mouse WB 1:1000
B56α Anti-B56α
Clone F-10 Santa Cruz sc-271151 Monoclonal Mouse WB 1:100
B56γ Anti-PP2A-B56γ, Clone E-6 Santa Cruz Biotechnology sc-374380 Monoclonal Mouse WB
1:100-500
PP2A-A Anti-PP2A-Aα/β Clone 4G7 Santa Cruz Biotechnology sc-13600 Monoclonal Mouse WB 1:250
Calnexin Purified Mouse Anti-Calnexin BD Transduction Laboratories™ 610523 Monoclonal Mouse WB
1:2000
Calnexin Anti-Calnexin antibody produced in rabbit Sigma
C4731 Polyclonal Rabbit WB 1:10000
ERC1 Anti-ERC1 [ELKS-30] Against residues 21-40 of ERC1a Abcam
ab50312 Monoclonal Mouse WB 1:1000
ERC1 Anti-ERC1 Sigma-Aldrich HPA019513 Polyclonal Rabbit IF 1:150
FLAG Monoclonal ANTI-FLAG® M2, clone M2 Sigma-Aldrich F1804 Monoclonal Mouse WB
1:1000
IF 1:500
GFP GFP Polyclonal Antibody Invitrogen A11122 Polyclonal Rabbit WB 1:2000
IP 2 µg
GFP Anti-GFP antibody Abcam ab13970 Polyclonal Chicken IF 1:1000
Lamins Anti-Lamin A + Lamin B1+ Lamin C Abcam Ab108922 Monoclonal Rabbit WB 1:5000
Liprin-α1 Anti-liprin-α1 (A-5) Santa Cruz Biotechnology sc-376141 Monoclonal Mouse IP 0.5 µg
IF 1:50
Liprin-α1 Anti-liprin-[illegible]1 Proteintech 14175-1-AP Polyclonal Rabbit WB 1:500
IF 1:150
Liprin-α1 Anti-liprin-[illegible]1; antigen: residues 818-1202 [6] Polyclonal Rabbit WB 1:500
Paxillin Purified Mouse Anti-Paxillin BD Transduction Laboratories™ 610052 Monoclonal Mouse WB
1:2000
IF 1:150
IP 2 µg
Paxillin Paxillin antibody GeneTex GTX125891 Polyclonal Rabbit IF 1:200
Src Clone 327 from S. Courtneidge [7] Monoclonal Mouse IF 1:50
pSrc Phospho-Src Family (Tyr416) Cell Signaling Technology #2101 Polyclonal Rabbit IF 1:100
Tubulin Monoclonal anti-α-Tubulin Sigma-Aldrich T9026 Monoclonal Mouse WB 1:4000
Vinculin Upstate Monoclonal Mouse
See complete list and details in the Methods section of the manuscript

Validation

When necessary, validation of antibodies including proper controls is described in the manuscript, or reference to previous characterization is provided.

Eukaryotic cell lines

Policy information about cell lines and Sex and Gender in Research

Cell line source(s)

COS7, MCF-7, NIH-3T3, HeLa, BT-474, SK-BR-3 and MDA-MB-231 from ATCC.

Authentication

MDA-MB-231 were either recently obtained from ATCC, or validated by IdentiCell STR allele report.

Mycoplasma contamination

We confirm that all cell lines used were negative for mycoplasma contamination.

Commonly misidentified lines
(See ICLAC register)

--
